# Genetic Structure and Hierarchical Population Divergence History of *Acer mono* var. *mono* in South and Northeast China

**DOI:** 10.1371/journal.pone.0087187

**Published:** 2014-01-31

**Authors:** Chunping Liu, Yoshiaki Tsuda, Hailong Shen, Lijiang Hu, Yoko Saito, Yuji Ide

**Affiliations:** 1 State Key Laboratory of Forest Genetics and Breeding, Northeast Forestry University, Harbin, Heilongjiang Province, China; 2 Laboratory of Forest Ecosystem Studies, Department of Ecosystem Studies, Graduate School of Agriculture and Life Science, The University of Tokyo, Tokyo, Japan; 3 Program in Plant Ecology and Evolution, Department of Ecology and Genetics, Evolutionary Biology Centre, Uppsala University, Uppsala, Sweden; Beijing Forestry University, China

## Abstract

Knowledge of the genetic structure and evolutionary history of tree species across their ranges is essential for the development of effective conservation and forest management strategies. *Acer mono* var. *mono*, an economically and ecologically important maple species, is extensively distributed in Northeast China (NE), whereas it has a scattered and patchy distribution in South China (SC). In this study, the genetic structure and demographic history of 56 natural populations of *A. mono* var. *mono* were evaluated using seven nuclear microsatellite markers. **N**eighbor-joining tree and STRUCTURE analysis clearly separated populations into NE and SC groups with two admixed-like populations. Allelic richness significantly decreased with increasing latitude within the NE group while both allelic richness and expected heterozygosity showed significant positive correlation with latitude within the SC group. Especially in the NE region, previous studies in *Quercus mongolica* and *Fraxinus mandshurica* have also detected reductions in genetic diversity with increases in latitude, suggesting this pattern may be common for tree species in this region, probably due to expansion from single refugium following the last glacial maximum (LGM). Approximate Bayesian Computation-based analysis revealed two major features of hierarchical population divergence in the species’ evolutionary history. Recent divergence between the NE group and the admixed-like group corresponded to the LGM period and ancient divergence of SC groups took place during mid-late Pleistocene period. The level of genetic differentiation was moderate (*F_ST_* = 0.073; *G′_ST_* = 0.278) among all populations, but significantly higher in the SC group than the NE group, mirroring the species’ more scattered distribution in SC. Conservation measures for this species are proposed, taking into account the genetic structure and past demographic history identified in this study.

## Introduction

Northeast China contains the country’s most important forest areas, but since the late 19th century forests in this region have been severely disturbed by wars, fires, deforestation, overexploitation and changes in land use to agriculture and grazing. Consequently, many tree species are now threatened [1]. Some conservation measures, such as designating areas as non-commercial forest zones and forbidding clear-cutting in degraded sites, have been applied in this region, but degradation of forests is still a serious threat [2]–[3]. The genetic diversity of historical lineages cannot be recovered if it is lost [4]. Thus, it is essential to understand the biological and genetic features of the tree species in a region’s forests [5]–[6] in order to formulate effective conservation and restoration programs that will maintain the diversity required to adapt to environmental changes [4], [9]–[10]. This requires robust assessments of the genetic diversity, structure and demographic history of species [7]–[8]. Further, the focus must be on preserving the overall genetic diversity and natural evolutionary processes in metapopulations rather than the diversity within separate subpopulations [11]. Thus, large-scale genetic structure analysis covering species’ ranges has been applied to numerous forest tree species, especially species that are economically, ecologically, and/or genetically important for a specific region [5]–[6], [8], [11]–[16].

The genetic diversity and population structure of various tree species in Northeast China have been evaluated using several types of molecular markers, for example *Phellodendron amurense* (AFLP) [17], *Quercus mongolica* (RAPD, AFLP and ISSR) [18]–[20], *Tilia amurensis* (ISSR) [21], and *Fraxinus mandshurica* (SSR) [14]. The genetic diversity of *Q. mongolica* [19] and *F. mandshurica* [14] has been shown to decrease with increasing latitude. Hu *et al.* [14] postulated that the detected cline in *F. mandshurica* populations arose from northward expansion of the forest biomes in the mid-Holocene from a single refugium located in eastern China during the last glacial maximum (LGM) [22]. This putative post-LGM population expansion to the north from southern refugium is believed to have shaped the modern genetic structure of many species in temperate zones in the northern hemisphere [23]–[24]. However, it is still not clear whether the decrease in genetic diversity with increasing latitude in the northern populations is a common pattern for tree species in Northeast China. Moreover, no coalescence-based genetic analysis has been previously applied to plant species in Northeast China, thus it is uncertain whether the reductions in genetic diversity are related to changes in species distribution driven by climate changes during the LGM. Recent forest genetics studies have suggested that an ancient demographic event predating the LGM has continuing effects on modern genetic diversity and structure [25]–[28]. Therefore, it is essential to evaluate the past population demography over sufficient time to elucidate the evolutionary history of a tree species and apply the acquired knowledge when formulating forest management and conservation strategies. Recently, a very powerful and flexible approach, Approximate Bayesian Computation (ABC), has been developed to estimate demographic and historical parameters (e.g. effective population size, divergence time) and quantitatively compare alternative scenarios [29]. This approach can be used to address many important questions in ecology and evolutionary biology [29].

**Figure 1:**
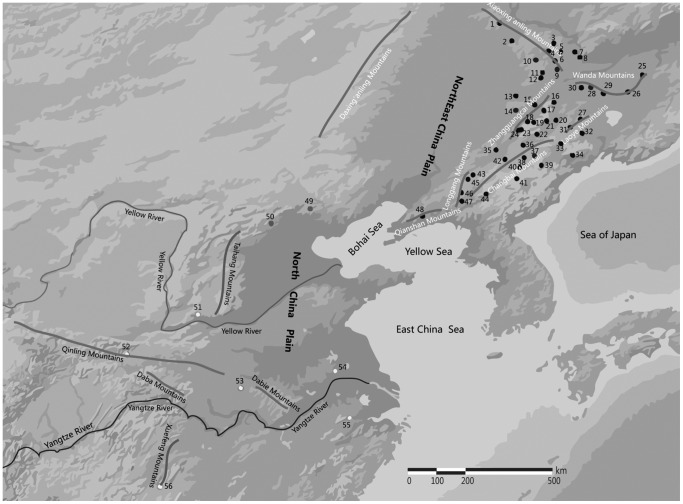
Locations of the 56 populations of *Acer mono* and geography of the study regions in China.

*Acer mono* (mono maple) is a common deciduous tree species that is widely distributed in east Asia, where there are many varieties [30]–[32], and an important companion tree species in the temperate mixed Korean pine and broadleaved forests of Northeast China [33]. It is an economically, ecologically, and culturally important species because of the unique properties of its wood, potential for production of sugar, oil, and medicinal materials, excellence for landscape planting and forestation of barren hills, and especially its attractiveness as an ornamental due to its vibrant red autumn foliage and fog-tolerance [34]. In China, *A. mono* is mainly distributed in Northeast China [32], but there are also scattered populations in forests of North China and the middle and lower reaches of the Yangtze River (Fig. 1). The natural resources of *A. mono* have decreased, and almost disappeared, at some sites due to: (1) harvesting of its wood for timber or fuel; (2) inappropriate forest management activities such as removal of all undergrowth species during thinning; and (3) of most importance recently, transplantation from natural forests to urban areas for use as ornamental, shade, or landscape trees. The conservation and sustainable management of natural populations of *A. mono*, as well as breeding and artificial planting of the species, are therefore now receiving more attention. However, few molecular ecology studies have addressed the genetic diversity and structure of natural populations of *A. mono* in China, although several have examined the reproduction and mating patterns of natural populations of the species in Japan [35]–[37].

We therefore conducted large-scale genetic analysis of *A. mono* using nuclear SSR markers to: 1) evaluate the wide-range genetic structure of 56 populations selected to cover its distribution in northeastern and southern China, 2) infer its population demographics using an ABC approach and 3) formulate conservation and management strategies for this natural genetic resource in Northeast China, taking into account its current genetic structure and inferred evolutionary history.

## Materials and Methods

### Ethics Statement

No specific permission was required for sampling activities in most populations because we sampled in the wild and only a few leaves were collected from each tree, except for some populations located in National Nature Reserves (listed after the corresponding populations in the “*Sampling and study sites*” section), in which the sampling activities were permitted by the relevant authorities. The field studies did not involve disturbance of any endangered or protected species.

### Sampling and Study Sites

We collected leaves of *Acer mono* var. *mono* from 48 natural populations, covering almost its entire distributions in Northeast China (hereafter NE populations), and eight in the rest of China (hereafter southern China, SC), where there are scattered, low-density populations (Fig. 1).

The NE populations were distributed in two major mountain ranges (numbers in brackets indicate assigned designations): (i) the Xiaoxing’anling Mountains (1–12), and (ii) the Changbai Mountains (13–48) including the main range (33–42, 44), branches such as the Zhangguangcailing (13–24) and Laoyeling Mountains (27, 31,32), and ranges extending outwards such as the Wandashan (25, 26, 28–30), Longgangshan (43, 45–47), and Qianshan Mountains (48) (Fig. 1). The individuals present at a location were treated as “a population”, a common approach in traditional genetic structure studies, thus several locations were examined within these ranges in NE. The SC populations are separated by much greater geographical distances than the NE populations, and their distributions are discontinuous because they are all located in separate mountain ranges: the Yanshan (49; Wulingshan National Nature Reserve, Hebei Province, China), Baihuashan (50), Zhongtiaoshan (51), Qinling (52), Jigongshan (53; Jigongshan National Nature Reserve, Henan Province, China), Langyashan (54; Langyashan National Nature Reserve, Anhui Province, China), Tianmushan (55; Tianmushan National Nature Reserve, Zhejiang Province, China), and Xuefengshan (56; Kanglong National Nature Reserve, Hunan Province, China) Mountains (Fig. 1). Individuals within each population were separated from each other by at least 30 m. In total, leaves from 1702 individuals were collected and stored in silica gel at room temperature until required for DNA extraction. The height and diameter at breast height of each individual were recorded when sampling.

### DNA Extraction and Genotyping

DNA was extracted using a modified CTAB method [38]. Nuclear SSR markers were selected from primers previously developed for Japanese *A. mono* [35] and other *Acer* species [39]–[41]. Seven of these markers (Am116, Am118, Am258, Am340, Am607 and Am742 [35], and Aca24 [39]) were found to be sufficiently stable and polymorphic and therefore used in this study.

Primers were mixed to a final concentration of 2 µM, except for primer Am258 (4 µM). The target sequences were then amplified in 5 µL reaction mixtures containing 2.5 µL of 2× Qiagen Multiplex PCR Master Mix, 0.5 µL of the primer mixture, 1.5 µL H_2_O, and 0.5 µL template DNA by PCR (95°C for 15 min, followed by 30 cycles of 94°C for 30 s, 58°C for 90 s, and 72°C for 60 s, with a final extension at 60°C for 30 min) using a TaKaRa TP600 Thermal Cycler. All PCR products were genotyped using an ABI 3130 Genetic Analyzer and the results were analyzed using GeneScan analysis software and Genotyper software version 3.5 (Applied Biosystems).

### Genetic Diversity within Populations

The FSTAT 2.9.3.2 software package [42] was used to evaluate the genetic diversity of each population by calculating the total number of alleles detected (*N_A_*), range of allele size (*R_AS_*), allelic richness (*A_R_*), expected heterozygosity (*H_E_*), and fixation index (*F_IS_*). In addition, significant deviations from Hardy-Weinberg equilibrium, as indicated by deviations of *F*_IS_ from zero, and genotypic disequilibrium for all locus pairs in each population, were tested by randomization using FSTAT. Rare allelic richness (*RA_R_*; [8]) and private allelic richness (*PA_R_*; [8]) were calculated. Pearson Correlation Analysis, implemented in SPSS 13.0 for Windows [43], was applied to determine possible correlations between genetic diversity parameters (*A_R_*, *RA_R_*, *PA_R_* and *H_E_*) and geographical gradients (of altitude, latitude and longitude). Since clear genetic differentiation was detected between the NE group (Populations 1–48) and the SC group (Populations 51–56) in the NJ tree and by STRUCTURE analysis (see *Results*), average values of *A_R_*, *H*_E_, *F*_IS_ and *F*_ST_ for each group and their differences between the two groups were tested using permutation tests in FSTAT. *RA_R_* and *PA_R_* of each group and the differences between the two groups were tested by One-way ANOVA using SPSS.

### Genetic Differentiation and Structure

The genetic differentiation among populations was evaluated by calculating *F*_ST_ values [44], and their significance was tested by comparison to 95% and 99% confidence intervals acquired from 1000 bootstraps. Pairwise *F*_ST_ values were also calculated, and the significance of pairwise population differentiation was tested by randomizing multilocus genotypes between the pairs using FSTAT. The source of genetic variation was determined by analysis of molecular variance analysis (AMOVA) [45], using GENALEx6.1 [46]. Standardized genetic differentiation (*G′_ST_*) was then calculated following Hedrick [47].

Isolation by distance (IBD) [48] was evaluated with GenAlEx 6.4 [46] using the method of Rousset (1997) [49], which tests for significant association between pairwise population differentiation (*F*_ST_/(1−*F*_ST_)) and the natural logarithms of direct minimum geographic distance between populations. The genetic relationships among populations were evaluated by generating a Neighbor-Joining (NJ) tree based on the *D*_A_ genetic distances [50], using Populations 1.2.30 beta software [51]. Statistical confidence in the topology of the tree was evaluated using 1000 bootstraps derived using the same software. The NJ tree was reconstructed on a topographic map using Mapmaker and GenGIS2 software packages [52]. Individual-based genetic structure was evaluated by Bayesian clustering as described by Pritchard*et al*. [53] using STRUCTURE 2.3.3 (hereafter, STRUCTURE analysis) [54]. We assumed a pre-assigned number of genetic clusters (*K*) ranging from 1 to 20. All runs involved 100,000 Markov Chain Monte Carlo (MCMC) generations, after a burn-in period of 100,000 iterations according to Hubisz *et al*. [55], based on the LOCPRIOR model described by Hubisz *et al*. [55], an admixture model and the correlated allele frequencies model (hereafter, the *F*-model) described by Falush *et al*. [56]. Ten runs were performed for each value of *K*. The optimum number of *K* was evaluated by calculating *ΔK* values [57] and applying the methods suggested in the original paper describing STRUCTURE analysis [53], which involves evaluating genetic structure by comparing mean log likelihoods of postulated models. Bar charts for the proportions of the membership coefficient of each individual in STRUCTURE analysis over 10 runs for each *K* were summarized using CLUMPP software [58] and visualized using DISTRUCT software [59].

### Population Demographic Analysis

**Figure 2:**
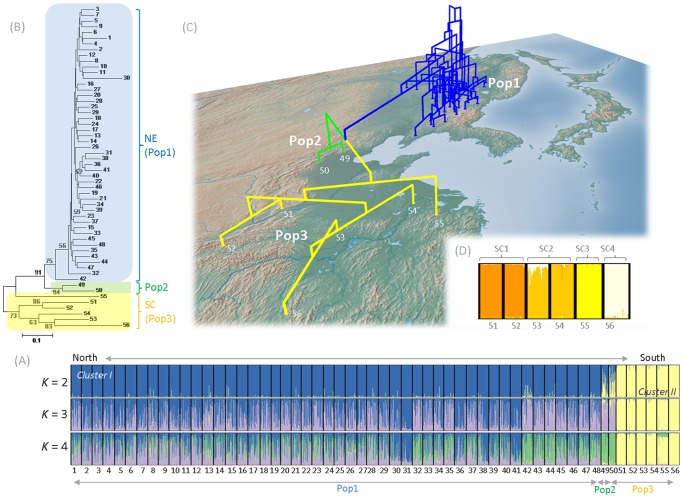
Results of STRUCTURE analysis and Neighbor-joining (NJ) tree of the 56 populations of *Acer mono* and STRUCTURE analysis of the South China populations. A, the proportion of the membership coefficient for each individual from 56 *A. mono* populations for the inferred clusters when *K* = 2 to 4 according to the STRUCTURE analysis; B and C, Neighbor-joining (NJ) tree of 56 populations based on *D*_A_ distance (Nei et al. 1983), showing bootstrap values exceeding 50%, definition of the three populations (Pop1, 2, and 3) used in DIYABC (A, B, C); D, the proportion of the membership coefficient for each individual from 6 SC populations for the inferred clusters when *K* = 4 according to the STRUCTURE analysis.

DIYABC v1.0.4.39 and v2.0 software [60]–[61] were used to infer the species’ demographic history by a two-step ABC approach. Populations covering the whole species’ distribution were first analyzed to infer the history of the topmost hierarchical genetic structure indicated by the STRUCTURE analysis (see *Results*, Fig. 2). Further ABC analysis then focused solely on SC populations as clear genetic structure was still detected among them in not only the STRUCTURE analysis but also the NJ tree (see *Results*, Fig. 2).

#### A) Demographic inferences across the whole species range

**Figure 3:**
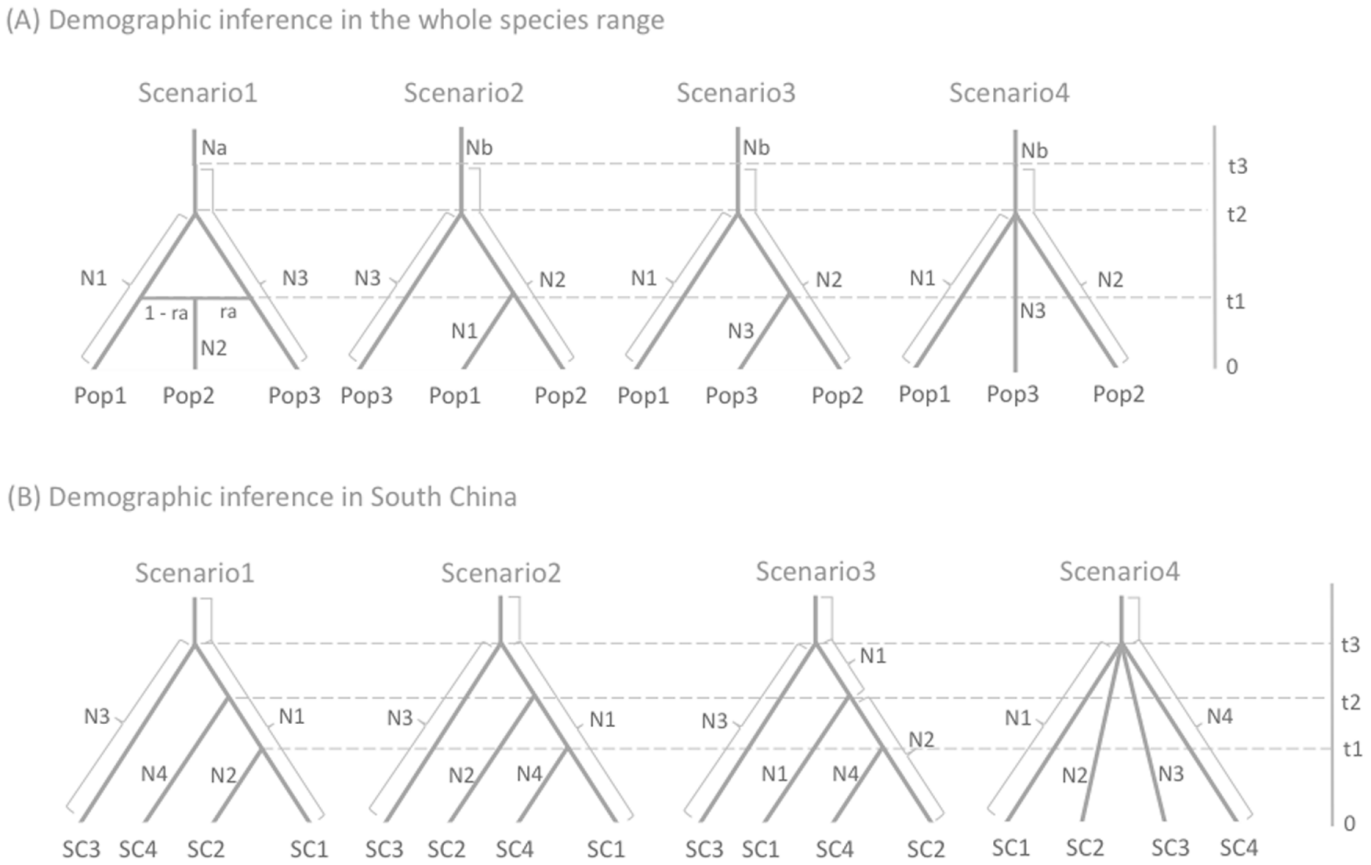
The four demographic scenarios for all populations (A) and South China populations (B) examined in DIYABC. t# is time scale measured in generations and N# is effective population size of the corresponding populations (A, Pop 1, 2, 3, a and b; B, SC1, 2, 3 and 4) during each time period (e.g. 0–t1, t1–t2, t2–t3).

To simplify scenarios for ABC analysis, we defined three populations based on the results of STRUCTURE analysis: Pop1 (Northeastern populations 1–48), Pop2 (admixed populations 49 and 50) and Pop3 (Southern populations 51–56). All sampled individuals of Pop2 (42 individuals) and Pop3 (177 individuals) were used for analysis. However, for Pop1, 200 individuals were randomly selected from the pool of 48 NE populations, as genetic structure among them was quite weak (*F_ST_* = 0.029, Fig. 2C) and use of all individuals (1483 in total) would have been computationally expensive without substantially increasing the amount of information acquired. We examined four simple population demography scenarios (Fig. 3A), taking into account the results of the NJ tree and the STRUCTURE analysis. In these scenarios, t# and N# refer to timescale (expressed as generation time) and effective size of the corresponding populations (Pops1, 2, 3 or the ancestral population) during each time period (e.g. 0– t1, t1– t2). The scenarios are as follows:

Scenario 1 *(isolation with admixture model):* Pop2 was generated by admixture of Pops 1 and 3 at t1, then Pop1 merged with Pop3 at t2. Based on the results of STRUCTURE analysis, this scenario was expected to be the most likely one.

Scenario 2 *(hierarchical split model 1):* Northern Pop1 merged with Pop2 at t1 and subsequently with southern Pop3 at t2.

Scenario 3 *(hierarchical split model 2):* Southern Pop3 merged with Pop2 at t1 and subsequently with Northern Pop1 at t2.

Scenario 4 *(simple split model):* all three populations diverged at the same time, t2.

In all scenarios, Pop2 was specified as the population to be traced back to an ancestral population (as required for DIYABC analysis). In addition, we assumed a bottleneck in the past at t3, which we positioned before the divergence point in all scenarios. We assumed past population growth and set the ancestral population size (Na in scenario 1 and Nb in scenarios 2–4) to be smaller than that of other populations. In DIYABC, we employed the higher mutation rate in the generalized stepwise mutation model (GSM) [62] with the lower mutation rate of single nucleotide indels (SNI) for modeling the mutation of SSRs. Default prior values were used for all these parameters (Table S1). The mean values of expected heterozygosity (*H_E_*) and number of alleles (*N_A_*) were used as the summary statistics for each of the three populations, while for summary statistics for each pair of populations from the three groups we used *H_E_*, *N_A_*, genotype likelihood and *F_ST_*. A million simulations were performed for each scenario. After the total of three million simulations, the most-likely scenario was evaluated by comparing posterior probabilities using logistic regression. The goodness of fit of the scenario was assessed by the option “model checking” with principal component analysis (PCA) in DIYABC, which measures the discrepancy between model and real data.

#### B) Demographic inferences in South China

Because the STRUCTURE analysis indicated that *ΔK* was highest at *K* = 4 and detected clear genetic structure (see *Results*, Fig. 2D), we defined four populations: SC1 (Pops 51 and 52), SC2 (Pops53 and 54), SC3 (Pop 55) and SC4 (Pop 56) to simplify demographic analysis of the SC populations. We examined the hierarchical split models (scenarios 1–3) and simple split model (scenario 4) using DIYABC v2.0 as shown in Fig. 3B. In the hierarchical split model, the most ancient split was set between SC3 and the others because SC3 was the most highly genetically differentiated of the SC populations according to the NJ tree (Fig. 2B). After the split from SC3, all permutations of pairs of SC1, 2 and 4 populations were set in the hierarchical split models in scenarios 1–3. The effective population sizes of SC1, 2, 3 and 4 were designated N1, N2, N3 and N4, respectively (setting N1> N2> N3> N4, according to their genetic diversity). Default prior values were used for all these parameters (Table S2), and the same approach was applied as for the whole-range analysis described above.

## Results

### Genetic Diversity of Populations

In total, 223 alleles were scored over the seven loci among the 1702 sampled *A. mono* individuals. The allelic diversity was highly variable across loci, with *N_A_* ranging from 14 to 45 (32±12 on average), *A_R_* from 4.824 to 16.778 (10.867±3.981 on average), and *H_E_* from 0.587 to 0.861 (0.737±0.110 on average) (Table 1). The fixation index (*F_IS_*) for individual loci ranged from –0.116 to 0.223 and the average over all loci was 0.053. No significant genotypic linkage disequilibrium for pairwise-loci was detected within any population (*P*>0.05).

**Table 1:** Genetic characteristics of the seven nSSR loci examined in all sampled individuals from 56 natural populations of *A. mono*.

Loci	*R_AS_* (bp)	*N_A_*	*A_R_*	*H_E_*	*F_ST_*
Aca24	90–113	14	4.824	0.587	0.088
Am116	236–298	45	16.778	0.833	0.077
Am118	153–189	18	7.301	0.610	0.121
Am258	164–259	39	9.628	0.740	0.071
Am340	164–242	36	12.120	0.702	0.072
Am607	130–180	27	11.997	0.861	0.035
Am742	150–203	44	13.424	0.829	0.060

Abbreviations: *R_AS_*, range of allele sizes; *N_A_*, number of alleles detected; *A_R_*, allelic richness; *H_E_*, expected heterozygosity.

The genetic diversity of each population is presented in Table 2. Values of *N_A_*, *R_A_*, *P_A_*, *A_R_*, *RA_R_*, *PA_R_* and *H_E_* ranged from 39 to 84, 8 to 55, 0 to 7, 5.181 to 11.210, 1.002 to 6.031, 0 to 1.293 and 0.535 to 0.840, respectively (Table 2). Although significant deviation of the *F*_IS_ value from zero was detected in 11 of 56 populations, there was no geographic pattern in the distributions of *F*_IS_ values (Table 2). When the correlations between genetic variation parameters (*A_R_* and *H_E_*) and geographical gradients (latitude, longitude and altitude) were evaluated, significant positive correlation was detected only between *H_E_* and latitude (*P*<0.05, Table 3). However, when geographical pattern of genetic diversity was analyzed within each of the NE and SC regions, different trends were detected: *A_R_* significantly decreased with increasing latitude within NE populations (*P*<0.01), while both *A_R_* and *H_E_* increased together with latitude within SC populations (*P*<0.05, Table 3).

**Table 2:** Locations and sizes of individuals in the 56 sampled populations of *Acer mono* and estimates of genetic diversity within populations.

Mountains			Locations	Lat (°N)	Long (°E)	Alt (m)	Height (m)	DBH (cm)	*N*	*N_A_*	*R_A_*	*P_A_*	*A_R_*	*RA_R_*	*PA_R_*	*H_E_*	*F_IS_*
Xiaoxing’anling		1	Heihe	49.590	126.786	460–480	6.2±2.3	9.30±4.32	29	43	8	0	5.992	1.002	0	0.684	–0.001
		2	Zhanhe	48.726	127.386	400–528	4.6±1.4	6.38±2.79	30	52	22	0	6.905	2.627	0	0.704	0.005
		3	Wuyiling	48.605	129.450	429–476	9.1±2.6	12.77±10.76	30	64	28	1	8.215	3.105	0.017	0.748	0.191*
		4	Wuying	48.234	129.201	366–430	11.0±2.8	12.81±6.54	33	65	35	0	7.964	3.691	0	0.693	–0.118
		5	Xinqing	48.193	129.780	455–550	14.0±2.5	19.90±7.16	30	60	24	0	7.791	2.705	0	0.703	–0.037
		6	Meixi	47.752	129.511	360–496	11.1±3.3	19.91±10.86	32	68	36	0	8.462	3.900	0	0.753	0.129*
		7	Hebei	48.187	130.483	234–388	13.3±2.8	15.49±7.48	31	67	37	1	8.325	4.043	0.016	0.735	0.009
		8	Luobei	47.917	130.731	199–226	11.1±3.1	11.80±4.67	29	69	28	0	9.071	3.245	0	0.790	0.246*
		9	Jinshantun	47.344	129.618	254–297	9.4±2.5	–	30	65	23	1	8.518	2.544	0.017	0.762	0.019
		10	Cuiluan	47.796	128.567	332–377	12.2±3.2	15.16±6.35	31	68	37	0	8.456	4.033	0	0.761	0.104
		11	Dailing	47.182	128.895	380–440	10.6±2.3	18.39±6.40	31	69	33	1	8.693	3.561	0.016	0.752	0.051
		12	Langxiang	46.922	128.821	337–418	10.7±2.1	18.36±8.09	31	65	36	0	8.430	4.292	0	0.743	0.075
Changbai	ZH	13	Mulan	46.051	127.585	75–108	10.8±2.8	17.94±8.54	32	76	44	1	9.198	4.638	0.016	0.762	0.075
		14	Maoershan	45.342	127.584	300–434	13.4±2.4	19.30±6.19	33	70	38	0	8.806	4.247	0	0.746	0.077
		15	Fangzheng	45.613	128.512	472–529	7.4±2.8	8.06±4.51	32	71	43	1	9.162	5.158	0.016	0.761	0.176*
		16	Baomashan	45.729	129.460	474–517	17.5±5.4	20.62±10.40	31	63	30	0	8.218	3.513	0	0.733	0
		17	Shuguang	45.339	128.958	107–233	10.3±3.1	20.33±10.70	34	76	38	1	9.331	3.931	0.015	0.765	0.149*
		18	Weihe	44.785	128.160	319–367	14.2±8.0	23.88±16.15	31	66	30	0	8.533	3.396	0	0.792	0.11
		19	Yabuli	44.761	128.465	467–526	13.3±7.8	10.83±7.85	26	62	27	0	8.469	3.477	0	0.745	0.019
		20	Chaihe	44.865	129.556	270–319	7.0±2.3	13.27±8.25	30	66	30	0	8.460	3.336	0	0.749	0.015
		21	Hailin	44.822	129.097	558–615	11.0±7.6	11.01±9.65	28	61	25	0	8.235	3.088	0	0.714	–0.105
		22	Ning’an	44.184	128.626	577–704	7.3±4.8	9.82±5.32	30	70	31	0	8.929	3.405	0	0.718	–0.068
		23	Shanhetun	44.402	127.854	290–451	15.9±4.2	19.33±8.67	32	84	55	1	10.168	6.031	0.016	0.792	0.081
		24	Shahe	44.369	127.705	322–363	10.8±3.2	24.66±11.14	30	72	28	1	9.143	2.892	0.017	0.779	0.096
	LY	27	Bamiantong	44.911	130.760	129–151	6.6±2.9	11.15±6.05	27	67	30	1	8.896	4.476	0.019	0.750	0.09
		31	Muling	44.518	130.262	96–151	8.5±2.8	14.13±6.85	31	52	32	0	8.486	3.497	0	0.535	–0.249
		32	Suiyang	44.225	130.847	402–466	11.0±2.6	17.07±8.69	29	71	35	1	8.685	3.409	0.017	0.792	0.085
	WD	25	Yingchun	47.051	133.800	241–327	13.7±4.5	15.26±7.80	31	70	39	1	9.987	5.426	0.016	0.752	0.063
		26	Dongfanghong	46.244	133.079	189–250	13.2±2.9	13.47±3.32	33	67	32	0	8.377	3.388	0	0.760	0.054
		28	Baoshan	46.471	131.268	108–154	14.2±3.9	18.72±11.26	33	83	51	0	7.687	2.849	0	0.776	0.135*
		29	Shuangyashan	46.161	131.880	333–592	14.1±4.2	15.60±7.78	31	65	30	0	6.411	3.559	0	0.756	0.141*
		30	Huanan	46.447	130.805	410–586	7.2±2.3	7.96±2.80	33	59	25	1	9.069	3.940	0.015	0.684	0.12
	CB	33	Wangqing	43.729	129.797	429–479	12.9±6.9	13.90±9.67	30	68	26	0	8.831	2.875	0	0.766	0.13*
		34	Hunchun	43.153	130.372	296–338	9.3±7.6	11.94±8.02	29	70	36	0	8.884	4.044	0	0.751	–0.109
		35	Yongji	43.417	126.613	554–617	11.6±5.3	17.87±9.69	30	74	34	0	9.384	3.705	0	0.777	0.069
		36	Huangnihe	43.647	127.942	469–534	13.4±5.9	19.64±10.08	33	57	27	0	7.045	2.771	0	0.716	–0.054
		37	Dashitou	43.127	128.503	616–660	10.3±3.0	12.02±4.56	31	69	40	0	8.660	4.524	0	0.768	0.107
		38	Dunhua	43.035	127.993	705–761	12.4±4.6	17.22±8.20	32	58	35	1	7.177	3.894	0.016	0.660	–0.109
		39	Helong	42.667	128.845	556–619	5.7±5.5	6.55±7.16	29	60	27	0	7.782	3.083	0	0.701	–0.012
		40	Lushuihe	42.554	127.779	735–771	12.1±6.2	16.66±10.35	31	75	47	1	9.344	5.348	0.016	0.742	–0.037
		41	Songjianghe	42.018	127.632	910–936	16.1±5.7	17.62±6.63	32	54	29	0	6.799	3.233	0	0.628	–0.081
		42	Hongshi	42.957	127.045	441–464	14.5±4.8	17.09±9.18	32	74	39	0	9.285	4.302	0	0.789	0.044
		44	Wunvfeng	41.268	126.122	473–586	12.5±4.5	11.82±5.02	31	84	44	1	11.007	5.307	0.016	0.840	0.078
	LG	43	Meihekou	42.196	125.480	459–503	12.9±4.4	18.81±9.18	32	73	38	0	9.241	4.252	0	0.807	0.148*
		45	Qingyuan	41.989	125.236	575–714	15.8±4.7	17.24±7.44	32	71	38	1	9.015	4.317	0.016	0.781	0.051
		46	Xinbin	41.331	124.913	673–821	13.7±4.6	16.70±7.05	31	73	46	0	9.211	5.357	0	0.749	–0.064
		47	Kuandian	40.920	124.927	496–634	11.5±3.0	14.25±4.92	32	84	50	0	10.287	5.442	0	0.821	0.190*
	QS	48	Xiuyan	40.183	122.998	320–406	7.5±2.6	12.90±4.97	32	75	42	1	9.432	4.724	0.016	0.809	0.122
Yanshan		49	Chengde	40.552	117.490	938–1029	6.0±2.8	8.67±6.34	21	58	28	1	8.286	4.000	0.024	0.755	0.062
Baihuashan		50	Beijing	39.836	115.576	1159–1264	7.9±2.7	8.93±3.55	21	58	29	5	8.286	4.143	0.703	0.710	0.176*
Zhongtiaoshan		51	Yuanqu	35.398	111.975	1409–1796	4.4±2.7	7.84±12.03	29	75	35	4	9.655	3.969	0.341	0.771	0.074
Qinling		52	Ningdong	33.443	108.479	1216–1842	5.3±1.8	4.48±2.93	27	86	44	7	11.210	5.216	1.293	0.818	0.039
Jigongshan		53	Lijiazhai	31.813	114.075	318–534	5.2±3.7	7.07±10.45	28	53	16	1	7.178	1.905	0.018	0.704	0.058
Langyashan		54	Chuzhou	32.077	118.806	100–200	10.6±6.2	12.47±10.14	30	50	15	1	6.579	1.592	0.017	0.680	0.016
Tianmushan		55	Lin’an	30.360	119.422	987–1095	4.3±1.8	5.73±1.89	33	55	27	1	6.902	2.918	0.015	0.550	0.079
Xuefengshan		56	Huaihua	27.017	110.104	503–1180	4.0±2.3	7.38±5.67	30	39	11	2	5.181	1.192	0.170	0.564	–0.022

Abbreviations: ZH, Zhangguangcailing Mts.; LY, Laoyeling Mts.; WD, Wandashan Mts.; CB, Changbai Mts.; LG, Longgangshan Mts.; QS, Qianshan Mts.; DBH, diameter at breast height; Lat, latitude; Long, longitude; Alt, altitude; *N*, number of individuals; *N_A_*, total number of alleles detected; *R_A_*, number of rare alleles; *P_A_*, number of private alleles; *A_R_*, allelic richness based on at least 21 diploid individuals of the smallest population; *RA_R_*, rare allelic richness based on 21 diploid individuals of the smallest population; *PA_R_*, private allelic richness based on 21 diploid individuals of the smallest population;*H_E_*, average expected heterozygosity; *F_IS_*, Wright’s inbreeding coefficient, where *indicates significant deviation from 0 when α = 0.05; –indicates missing data.

**Table 3:** Pearson Correlation Coefficients between genetic variation and geographical gradients.

	All populations		NE populations		SC populations	
	*A_R_*	*H_E_*	*A_R_*	*H_E_*	*A_R_*	*H_E_*
Latitude	0.133	0.289*	–0.416**	–0.225	0.814*	0.844*
Longitude	0.07	0.154	–0.218	–0.24	–0.463	–0.491
Altitude	0.05	–0.115	–0.159	–0.145	0.726	0.406

Values indicate correlation indices, * and **indicate significant correlation when α = 0.05 and 0.01, respectively.

### Population Genetic Structure

The *F_ST_* among all populations was 0.073, with 95% and 99% confidence intervals (CI) ranging from 0.056 to 0.093 and 0.051 to 0.1, respectively, and 66.35% of pairwise-*F_ST_*values were significant (*P*<0.05). The overall value of *F_ST_* corresponded to a standardized genetic differentiation (*G′_ST_*) of 0.278, suggesting moderate population structure. Separate analysis of the genetic variation in NE and SC populations, based on results of the STRUCTURE analysis and the NJ tree (see below), showed that *H_E_* and *RA_R_* values were significantly higher for NE than SC population (*H_E = _*0.743 and 0.676, *RA_R_* = 3.825 and 2.799, respectively; Table 4). In contrast, *PA_R_* and *F_ST_* values were significantly higher for SC than NE populations (*PA_R_* = 0.309 and 0.006, *F_ST_* = 0.207 and 0.029, respectively), but no significant differences in *A_R_* was detected (Table 4). The *F*_IS_ values of both groups were almost zero (0.052 in NE and 0.042 in SC) and there was no significant differentiation between them (Table 4). The *G′_ST_* values for the NE and SC groups were 0.114 and 0.722, respectively, suggesting strong genetic structure in the SC group. Thus, 96.4% of the pairwise-*F_ST_* values were significant (*P*<0.05) in the SC group while the proportion was much lower (56.2%) in the NE group. Moreover, separate analysis of the pattern of genetic diversity within the groups showed that genetic variation decreased with increasing latitude in NE populations, but increased in SC populations (Table 3). Analysis of molecular variance based on these two groups showed that within-population variation, variation among populations in each region and between-region variation accounted for 76%, 16% and 8% of the total detected genetic variation, respectively (*P* = 0.01). Mantel tests indicated that there was significant isolation by distance in all populations (*R^2^* = 0.4241,*P* = 0.000), but not within either the NE or SC populations (*R^2^* = 0.009,*P* = 0.090 for NE populations; *R^2^* = 0.081,*P* = 0.060 for SC populations).

**Table 4:** Comparison of genetic diversity parameters: allelic richness (*A_R_*), rare allelic richness (*RA_R_*), private allelic richness (*PA_R_*), fixation index (*F_IS_*), averaged expected heterozygosity (*H_E_*), and Weir & Cockerham’s *F_ST_* (1984) between the Northeastern (NE) and the Southern (SC) group.

Group	*A_R_*	*RA_R_*	*PA_R_*	*F_IS_*	*H_E_*	*F_ST_*
NE (PopulationNos. 1–48)	8.59	3.825	0.006	0.052	0.743	0.029
SC (PopulationNos. 51–56)	7.78	2.799	0.309	0.042	0.676	0.207
*P*-value	0.104	0.024	0.000	0.789	0.009	0.002

According to the whole-range STRUCTURE analysis, the probability of the LnP(D) data increased progressively with increases in *K* up to *K* = 8, where it started to plateau (Fig. S1). However, the results for *K* >4 showed complex admixture patterns and none of the clusters were specific to any populations or regions. Since Δ*K* indicated that the optimal value for *K* was 2 (Fig. S1), in the following text discussion of population genetic structure is mainly based on results acquired with *K* = 2. In this case, all NE populations clustered into one group, and populations 51–56 clustered into the other group. Populations 49 and 50, located in Northern China, adjacent to the regions occupied by both groups, appeared to possess admixed-like features of the two groups (Fig. 2A, *K* = 2). However, with values of *K* = 3 and 4, although genetic structure was not clear over all NE populations, these two populations were assigned to a specific cluster. The NJ tree showed similar genetic structure to that detected by the STRUCTURE analysis, with two main groups corresponding to the NE and SC populations (Fig. 2B, C). Populations 49 and 50, for which STRUCTURE analysis suggested admixture when *K* = 2, fell between the two groups (Fig. 2B, C). In addition, most of the nodes in the NE populations were poorly supported by the bootstraps due to weak population structure, but most of the nodes for Populations 49 and 50, and SC populations, were supported by higher bootstrap values (Fig. 2B), indicating strong genetic structure. Because of higher differentiation, STRUCTURE analysis on the 6 populations in SC was conducted. These six populations divided into four clusters, according to the highest *ΔK* (Fig. 2D) [57], and when *K* = 6 they were clearly divided into six population-specific clusters (Fig. S2A, *K* = 6). Moreover, separate STRUCTURE analysis of NE populations detected weak genetic structure among populations associated with the mountain ranges, notably the 12 populations located in the Xiaoxing’anling Mountains (1–12) were almost entirely dominated by a single cluster (Fig. S2B, *K* = 2).

### ABC-based Inferences of Population Demography

In DIYABC analysis, based on the whole range STRUCTURE analysis, scenario 1, (the isolation with admixture model) was expected to be the most likely. However, the highest posterior probability was found for scenario 2 (the hierarchical split model 1), and the value (0.7341, 95% CI: 0.7079–0.7602) was much higher than those for scenario 1 (0.2557), 3 (0.0032) and 4 (0.0071) (Table 5A). In scenario 2, the median values of effective population size of N1 (Pop1), N2 (Pop2), N3 (Pop 3), and Nb (ancestral population) were 6570, 3700, 8700 and 1470, respectively (Table S3). Estimated median times of recent divergence (t1 for Pop 1 and Pop2) and ancient divergence (t2 for southern Pop3 and the others) were 681 and 2960 generations ago, respectively. Assuming the generation time of *A. mono* to be 30 years, the divergence times of t1 and t2 correspond to 20,430 and 88,800 years ago, respectively. The median time for a pronounced increase in population size (t3) was estimated at 6480 generations ago (194,400 years with a generation time of 30 years). However, the posterior distribution pattern suggested that this estimate was not robust (Fig. S3). Estimated median mean mutation rates of SSR and SNI were estimated to be 4.80×10^−4^ and 8.41×10^−5^, respectively (Table S3). None of the expected heterozygosity (*H_E_*) and number of alleles (*N_A_*) values for any population – or *H_E_*, *N_A_*, genotype likelihood and *F*_ST_ values for any population-pair – estimated using the acquired posterior distributions significantly differed from observed values, suggesting that scenario 2 was a good fit (Table S4). This was corroborated by the PCA, which yielded a large cloud of data from the prior and observed datasets, centered around a small cluster from the posterior predictive distribution (Fig. S4).

**Table 5:** Posterior probability of each scenario and the corresponding 95% confidence interval based on logistic estimation by DIYABC.

Scenario	Posterior probability	95% CI (lower − upper)
A) Analysis across the whole range		
1	0.2557	0.2297–0.2817
2	0.7341	0.7079–0.7602
3	0.0032	0.0022–0.0041
4	0.0071	0.0055–0.0087
B) Analysis of South China (SC) populations		
1	0.0247	0.0190–0.0305
2	0.0272	0.0211–0.0333
3	0.9262	0.9140–0.9384
4	0.0219	0.0165–0.0272

The DIYABC based on SC populations STRUCTURE analysis of the SC populations (*K* = 4) found the highest posterior probability for the hierarchical population split scenario 3 (0.9262, 95% CI: 0.9140-0.9384; Table 5B), in which the median values of effective population size of N1 (SC1), N2 (SC2), N3 (SC3) and N4 (SC4) were 8930, 3890, 2390 and 866, respectively (Table S5). The median estimated values of time of t1, t2 and t3 were 651, 2960 and 6330 generations ago, respectively (Table S5), while the median estimated mean mutation rates of SSR and SNI were 6.29×10^−4^ and 5.80×10^−7^, respectively (Table S5). The posterior probability (Fig. S5), summary statistics (Table S6) and the PCA results (Fig. S6) all suggested that scenario 3 provides a good fit to the data.

## Discussion

### Genetic Diversity of *A. mono* Populations

In Northeast China, although the forest zone is threatened, nSSR studies have shown that several tree species maintain moderate to strong genetic diversity, similar to that of *A. mono* (*H_E_* = 0.738±0.058), e.g. *Fraxinus mandshurica* (*H_E_* = 0.746±0.051) [14] and *Juglans mandshurica* (*H_E_* = 0.756) [63]. Significant positive correlations between genetic diversity (*H_E_*) and latitude was detected over all the 56 populations (Table 3). However, as both *A_R_* and *H_E_* showed significant positive correlation with latitude in SC group (Table 3), the smaller population size and the greater geographical distances between populations in SC than in NE may have strongly contributed to this overall pattern. Thus, different trends were detected within NE populations and *A_R_* values significantly decreased with increasing latitude. Although it was not significant, *H_E_* also showed the similar negative correlation with latitude. As mentioned in the Introduction, the reductions in genetic diversity with increasing latitude observed in NE populations of *Q. mongolica* [19] and *F. mandshurica* [14] were also found in *A. mono*, indicating that it may be a common trend for tree species in this region. According to previous studies [64], the genetic diversity of most species declines from central to marginal populations. We therefore inferred a probable route for the expansion of *A. mono*, from the central section of China toward the north and south. However, the lower diversity, especially the low rare allelic richness in populations 1 and 53–56, may not be mainly due to margin effects. For population 1, extremely low winter temperature may contribute to low genetic diversity by hindering the growth of seedlings and young saplings [14], or even killing them, thereby reducing the population size and rare allelic richness. For populations 53–56, besides the patchy and scattered distribution of the species in their region, another factor contributing to lower rare allelic richness may be landscape fragmentation (one of the major factors causes of dramatic loss of biodiversity globally) by blocking gene flow among populations, altering the biogeographical environments and diminishing available habitats [65].

Although inbreeding will increase homozygosity in the short term and affect diversity in the longer term [66], neither *H_E_* nor *A_R_* in the 11 populations for which *F*_IS_ values significantly deviated from 0 (Table 2) were significantly lower than others. Thus, reduction of genetic diversity induced by inbreeding was not suggested in this study. Instead of inbreeding, demographic factor such as Wahlund effect would be better explanation to higher values of F*_IS_* detected in this study.

### Genetic Structure and Demographic History of *A. mono* Populations

The *F_ST_* and *G′_ST_* values indicated that differentiation was much lower among NE populations (*F_ST_* = 0.029; *G′_ST = _*0.114) than among SC populations (*F_ST_* = 0.201; *G′_ST = _*0.722). For the NE populations, the relatively short geographic distances between populations and the continuous distribution of mountains provide a more uniform landscape that probably facilitated gene exchange among populations [69], explaining the higher diversity and lower differentiation observed. The weak genetic structure and reductions in genetic diversity with latitude among the NE populations suggest that they expanded from a single lineage. Interestingly, similar observations (no significant substructure and latitudinal reduction of genetic diversity among mountain ranges in Northeast China) have also been reported for an ash species, *F. mandshurica* [14]. The patterns of both species could be explained by reduction of genetic diversity to the north due to expansion from a single refugium in the last ice age. STRUCTURE analysis focused solely on NE populations detected weak genetic structure among mountain ranges (Fig. S2B, *K* = 2). More specifically, the clustering pattern shown by the barplots indicated that the 12 populations located in the Xiaoxing’anling Mountains (1–12) were almost entirely dominated by a single cluster. Thus, the weak genetic structure in NE populations was probably due to the mountain ranges limiting gene flow, as they often do for tree species [70]. Since *A. mono* is an insect-pollinated species [71], and its large winged seeds are dispersed by wind, the potential for gene flow may be more sensitive to geographic barriers than that of wind-pollinated and wind-dispersed species such as birch (*Betula maximowicziana*), which was examined by Tsuda *et al*. [70]. In addition, the STRUCTURE analysis and NJ tree separated all populations into three groups (NE and SC populations, and those from adjacent regions). The analysis indicated that the two populations (49 and 50) located in northern China were admixed by STRUCTURE analysis when *K* = 2, but they appeared as an additional, independent genetic group in the results with *K* = 3 and 4. Since the isolation by admixture scenario was not supported by the ABC analysis, we propose that these two populations constitute an independent group.

The demographic history of plant species associated with the LGM and species refugia in Northeastern China has been discussed [14], [63], but no coalescent-based genetic analysis has been previously applied. Thus, the results of ABC analysis obtained in this study provide valuable foundations for addressing the demographic history of *A. mono* over a long time scale. The estimated timing of t3 (a median value of 6480 generations, corresponding to 194,400 years) was not robust (Fig. S3), but since the effective population size increased to 3700 (N2) from 1470 (Nb) at t3, the results clearly indicate that the population expanded rapidly at or around this time.

The ancient divergence of Pop3 at t2, may have been caused by the Taihang Mountains acting as a barrier. These Mountains have a north-south orientation (36–40°N, 112–115°E) and form a major natural boundary in eastern China. The southern Taihang Mountain marginal fault zone has been a major boundary of neotectonic deformation since the mid-late Pleistocene, dividing northern China latitudinally, and the south region containing the deformation system that includes the south Qinling Mountains [72]. This division might explain not only the distribution of cpDNA haplotypes of *Juglans mandshurica* in the south and north of the Taihang and Qinling Mountains (Fig. 1) [63], but also the divergence of Pop3. In further support of this hypothesis, maternally-inherited mtDNA patterns of *Pinus tabulaeformis* are reportedly divided into three geographical groups, separated latitudinally by the Yellow River and longitudinally by the Taihang Mountains [12]. Pop1 and Pop2 were separated by the Northeast Plain, the largest Plain in China with a total area of 350,000 km^2^ (40° 25′ to 48° 40′N, 118°40′ to 128°E), and their estimated divergence time was estimated at 20,430 a BP. The Northeast Plain was covered by coniferous forest/steppe during the early late Pleistocene and broad-leaved forest during the middle late Pleistocene, but at the end of the Pleistocene (42,000 ∼ 20,300 a BP), because of the colder and drier climate, most of the Northeast Plain was covered by sand, and the vegetation changed to open forest/steppe [73]. Thus, Pop1 and 2 appear to have diverged during this period, when the species occupied refugia before the LGM.

STRUCTURE analysis divided SC populations (Pop3) into four subgroups. The strong genetic structure in these populations was probably caused by a combination of the greater geographic distances separating them [16], [67] and the presence of geographic barriers such as discontinuous mountain ridges [68]–[69] and large plains and valleys [25] (Fig. 1). These factors presumably isolated the populations and thus promoted population differentiation by limiting the potential for gene exchange. DIYABC analysis of the SC populations provided estimated divergence time (t1, t2 and t3) of each subgroup (SC1, SC2, SC3 and SC4) but the estimate for t3 (189,900 years) was not robust (Fig. S5). SC2 and SC4 apparently diverged from SC1 at 88,800 a BP (t2), possibly due to a major uplift of the Qinling Mountains. These mountains form a major boundary between north and south China, ranging west-east (32–35°N, 103–113°E) and rose strongly during the Quaternary, especially the Late Quaternary (100,000 a BP), when they reportedly rose up to 5.4 mm per year [74]–[75]. Thus, if we accept t3 as accurate, we can assume that the Qinling Mountains rose several tens or hundreds meters during the t3 to t2 period, which could have been a major contributor to the divergence of SC1 and SC2.

### Implications for Genetic Breeding, Conservation, and Silviculture of *A. mono*

A shelter wood system of forest management has been found to have little or no impact on genetic diversity and mating in *Fagus sylvatica* [76]. However, extensive logging can significantly alter the level and distribution of genetic variation in *Acer saccharum* [77]. The cited studies indicate that silvicultural activities may affect genetic diversity, but in ways that vary according to species and management type.

Natural resources of *A. mono* in China are under increasing pressure due to its use as a source of wood and various other materials. Hence, specific natural varieties are being selected for artificial planting and silviculture in Northeast China and elsewhere. We only used neutral genetic markers in the present study, but the information obtained is potentially highly informative for the breeding, conservation and silviculture of *A. mon*o, especially for the designation of conservation units. Since clear genetic differentiation was found between NE and SC populations, these two groups should be treated as independent conservation units. Although admixture was found for Populations 49 and 50 in the STRUCTURE analysis when *K* = 2, the ABC-based analysis suggested that these two populations were not generated by the admixture of NE and SC populations. Thus, populations in this region of northern China located between NE and SC populations should also be treated as an independent conservation unit. Seeds and nursery stocks of local provenance should be used in reforestation efforts in each area to avoid nonlocal introductions and genetic homogenization [11], [78]. Moreover, since strong genetic structure was detected in SC populations, further local-scale studies in this region are needed to develop appropriate strategies for conservation and the designation of natural reserves. In addition, *ex situ* conservation efforts, which could involve collecting seeds from populations in this area, may be useful. Since Populations 51 and 52 showed high levels of diversity and more private alleles than other populations, natural reserve areas should be established in these regions. In contrast, the NE populations maintain relatively high levels of genetic diversity and densities of individuals within populations, but low genetic differentiation among populations, implying that the species should be able to recover without assistance (if further disturbance is avoided) in this region. Selection of elite trees and the development of varieties for specific purposes are likely to be more important than provenance selection for conserving *A. mono* in Northeast China. To obtain the thorough overview required to develop an effective conservation strategy for this species further studies are required, including evaluation of the extent of its adaptive genetic variation.

## Supporting Information

Figure S1 Mean values of ln P(D) and standard deviations obtained from 10 runs for each value of *K* = 1–20, and distributions of *ΔK* (Evanno et al. 2005) for *K* = 1–20.
(TIF)

Figure S2 Results of additional runs of STRUCTURE analysis focused on the six SC and 48 NE populations. A, *K* = 6, in which the clustering corresponded to each of the six SC populations; B, *K* = 2 (according to the highest *ΔK*) for the 48 NE populations.
(TIF)

Figure S3 Prior and posterior distributions for each parameter obtained by DIYABC analysis of populations across the whole range.
(TIF)

Figure S4 Principal Component Analysis (PCA) score plot obtained from DIYABC analysis of populations across the whole range.
(TIF)

Figure S5 Prior and posterior distributions for each parameter obtained by DIYABC analysis of in South China populations.
(TIF)

Figure S6 Principal component analysis (PCA) score plot obtained from DIYABC of South China populations.
(TIF)

Table S1 Prior distributions of the parameters used in the whole-range DIYABC analysis.
(DOC)

Table S2 Prior distributions of the parameters used in the South China DIYABC analysis.
(DOC)

Table S3 Demographic parameters obtained from the whole-range DIYABC analysis.
(DOC)

Table S4 Comparison of summary statistics for the observed data set and posterior simulated data sets.
(DOC)

Table S5 Demographic parameters obtained from DIYABC analysis of South China populations.
(DOC)

Table S6 Comparison of summary statistics for the observed data set and simulated posterior data sets.
(DOC)

## References

[1] Fu LG (1992) China Plant Red Data Book, Rare and Endangered Plants (Vol. 1). Beijing: Chinese Science Press. 385–387p (in Chinese).

[2] Li WH (2004) Degradation and restoration of forest ecosystems in China. For Ecol Manage 201: 33–41.

[3] Zhu JJ, Mao ZH, Hu LL, Zhang JX (2007) Plant diversity of secondary forests in response to anthropogenic disturbance levels in montane regions of northeastern China. J For Res 12: 403–416.

[4] Moritz C (2002) Strategies to protect biological diversity and the evolutionary process that sustain it. Syst Biol 51: 238–254. 12028731 10.1080/10635150252899752

[5] Escudero A, Iriondo JM, Torres ME (2003) Spatial analysis of genetic diversity as a tool for plant conservation. Biol Conserv 113: 351–365.

[6] Newton AC, Allnutt TR, Gillies ACM, Lowe AJ, Ennos RA (1999) Molecular phylogeography, intraspecific variation and the conservation of tree species. Trends Ecol Evol 14: 140–145. 10322519 10.1016/s0169-5347(98)01555-9

[7] Geburek T (1997) Isozymes and DNA markers in gene conservation of forest trees. BiodiversConserv 6: 1639–1654.

[8] Tsuda Y, Ide Y (2005) Wide-range analysis of genetic structure of *Betula maximowicziana*, a long-lived pioneer tree species and noble hardwood in the cool temperate zone of Japan. Mol Ecol 14: 3929–3941. 16262849 10.1111/j.1365-294X.2005.02715.x

[9] Crandall KA, Bininda-Emonds ORP, Mace GM, Wayne RK (2000) Considering evolutionary processes in conservation biology. Trends EcolEvol 15: 290–295. 10.1016/s0169-5347(00)01876-0 10856956

[10] Frankel OH (1974) Genetic conservation: our evolutionary responsibility. Genetics 78: 53–65. 17248668 10.1093/genetics/78.1.53 PMC1213213

[11] Steinitz O, Robledo-Arnuncio JJ, Nathan R (2012) Effects of forest plantations on the genetic composition of conspecific native Aleppo pine populations. Mol Ecol 21: 300–313. 22151559 10.1111/j.1365-294X.2011.05394.x

[12] Chen K, Abbott RJ, Milne RI, Tian XM, Liu J (2008) Phylogeography of *Pinus tabulaeformis* Carr. (Pinaceae), a dominant species of coniferous forest in northern China. Mol Ecol 17: 4276–4288. 19378405 10.1111/j.1365-294x.2008.03911.x

[13] Heuertz M, Hausman JF, Hardy OJ, Vendramin GG, Frascaria-Lacoste N, et al (2004) Nuclear microsatellites reveal contrasting patterns of genetic structure between Western and Southeastern European populations of the common ash (*Fraxinus excelsior* L). Evolution 58: 976–988. 15212379 10.1111/j.0014-3820.2004.tb00432.x

[14] Hu LJ, Uchiyama K, Shen HL, Saito Y, Tsuda Y, et al (2008) Nuclear DNA microsatellites reveal genetic variation but a lack of phylogeographical structure in an endangered species, *Fraxinus mandshurica*, across North-east China. Ann Bot 102: 195–205. 18477559 10.1093/aob/mcn074 PMC2712365

[15] Hu LJ, Uchiyama K, Shen HL, Saito Y, Ide Y (2010) Multiple-scaled spatial genetic structures of *Fraxinus mandshurica* over a riparian-mountain landscape in Northeast China. Conserv Genet 11: 77–87.

[16] Logossa ZA, Camus-Kulandaivelu L, Allal F, Vaillant A, Sanou H, et al (2011) Molecular data reveal isolation by distance and past population expansion for the shea tree (*Vitellariaparadoxa* C.F. Gaertn) in West Africa. Mol Ecol 20: 4009–4027. 21914014 10.1111/j.1365-294X.2011.05249.x

[17] Yan ZF, Zhang BG, Zhang Z, Yu JL (2006) Genetic diversity in wild populations of *Phellodendron amurense*, a rare and endangered medicinal plant, detected by AFLP. Chinese Biodiversity Science14: 488–497 (in Chinese with English abstract)

[18] Xia M, Zhou XF, Zhao SD (2001) RAPD analysis on genetic diversity of natural populations of *Quercus mongolica* . Scientia Silvae Sinicae 37: 126–133 (In Chinese with English abstract)

[19] Zhang J, Wu D, Wang CL, Qu HJ, Zhou XZ, et al (2007) Genetic diversity analysis of *Quercus mongolica* populations with Inter-Simple Sequence Repeats (ISSR) technique. Biodiversity Science 15: 292–299 (in Chinese with English abstract)

[20] Li YW, Gu WC, Zhou SL (2003) AFLP analysis on genetic diversity of *Quercus mongolica* populations. Scientia Silvae Sinicae 39: 29–36 (in Chinese with English abstract)

[21] Mu LQ, Liu YN (2007) Genetic diversity of *Tulia amurensis* populations indifferent geographical distribution regions. J Plant Ecol (Chinese Version) 31: 1190–1198 (in Chinese with English abstract)

[22] Yu G, Prentice IC, Harrison SP, Sun X (1998) Pollen-based biome reconstructions for China at 0 ka and 6 ka. J Biogeogr 25: 1055–1069.

[23] Hewitt G (2000) The genetic legacy of the Quaternary ice ages. Nature 405: 907–913. 10879524 10.1038/35016000

[24] Hewitt G (2004) Genetic consequences of climatic oscillations in the Quaternary. Philos Trans R Soc Lond B Biol Sci 359: 183–195. 15101575 10.1098/rstb.2003.1388 PMC1693318

[25] Magri D, Vendramin GG, Comps B, Dupanloup I, Geburek T, et al (2006) A new scenario for the Quaternary history of European beech populations, palaeobotanical evidence and genetic consequences. New Phytol 171: 199–221. 16771995 10.1111/j.1469-8137.2006.01740.x

[26] Magri D, Fineschi S, Bellarosa R, Buonamici A, Sebastiani F, et al (2007) The distribution of *Quercus suber* chloroplast haplotypes matches the palaeogeographical history of the western Mediterranean. Mol Ecol 16: 5259–5266. 17995923 10.1111/j.1365-294X.2007.03587.x

[27] Lascoux M, Pyhäjärvi T, Källman T, Savolainen O (2008) Past demography in forest trees: what can we learn from nuclear DNA sequences that we do not already know? Plant Ecol Divers 1: 209–215.

[28] Ingvarsson PK (2008) Multilocus patterns of nucleotide polymorphism and the demographic history of *Populus tremula* . Genetics 180: 329–340. 18716330 10.1534/genetics.108.090431 PMC2535685

[29] Bertorelle G, Benazzo A, Mona S (2010) ABC as a flexible framework to estimate demography over space and time: some cons, many pros. Mol Ecol 19: 2609–2625. 20561199 10.1111/j.1365-294X.2010.04690.x

[30] Hsu T (1992) Variation patterns and systematics of *Acer mono* Maxim.Guihaia. 12: 229–234 (in Chinese with English abstract)

[31] Satake G, Hara K, Hara H, Watari S, Tominali U (1993) Wild flowers of Japan: woody plants. Tokyo: Heibonsha (in Japanese).

[32] Zheng W (2004) Sylva Sinica (vol. 4). Beijing: Chinese publishing House of Forestry. 4258–4260p (in Chinese).

[33] Dong SL (1985) Study on Aceraceae of northeastern China. Bulletin of Botanical Research 5: 97–111 (in Chinese)

[34] Hsu T (1988) Evaluation on *Acer* plant resources in China. Resource Development & Market 4: 51–54.

[35] Kikuchi S, Shibata M (2008) Development of polymorphic microsatellite markers in *Acer mono* Maxim.Mol Ecol Resour. 8: 339–341. 10.1111/j.1471-8286.2007.01948.x 21585785

[36] Kikuchi S, Shibata M, Tanaka H, Yoshimaru H, Niiyama K (2009) Analysis of the disassortative mating pattern in a heterodichogamous plant, *Acer mono* Maxim. using microsatellite markers. Plant Ecol 204: 43–54.

[37] Shibata M, Kikuchi S, Tanaka H, Sueyoshi M, Yoshimaru H, et al (2009) Effects of population density, sex morph, and tree size on reproduction in a heterodichogamous maple, *Acer mono*, in a temperate forest of Japan. Ecol Res 24: 1–9.

[38] Lian CL, Oishi R, Miyashita N, Nara K, Nakaya H, et al (2003) Genetic structure and reproduction dynamics of *Salix reinii*during primary succession on Mount Fuji, as revealed by nuclear and chloroplast microsatellite analysis. Mol Ecol 12: 609–618. 12675817 10.1046/j.1365-294x.2003.01756.x

[39] Terui H, Lian CL, Saito Y, Ide Y (2006) Development of microsatellite markers in *Acer capillipes* . Mol Ecol Notes 6: 77–79.

[40] Segarra-Moragues JG, Gleiser G, González-Candelas F (2008) Isolation and characterization of microsatellite loci in *Acer opalus* (Aceraceae), a sexually-polymorphic tree, through an enriched genomic library. Conserv Genet 9: 1059–1062.

[41] Pandey M, Gailing O, Fischer D, Hattemer DH, Finkeldey R (2004) Characterization of microsatellite markers in sycamore (*Acer pseudoplatanus*L.). Mol Ecol Notes 4: 253–255.

[42] Goudet J (2001) FSTAT, a program to estimate and test gene diversities and fixation indices (ver. 2.9.3). Available: http://www2.unil.ch/popgen/softwares/fstat.html.

[43] SPSS Inc. (2004) SPSS for Windows, Release 13.0, September 1, Chicago, IL, SPSS Inc.

[44] Weir BS, Cockerham CC (1984) Estimating *F*-Statistics for the analysis of population structure. Evolution 38: 1358–1370. 28563791 10.1111/j.1558-5646.1984.tb05657.x

[45] Excoffier L, Smouse PE, Quattro JM (1992) Analysis of molecular variance inferred from metric distances among DNA haplotypes: application to human mitochondrial DNA restriction data. Genetics 131: 479–491. 1644282 10.1093/genetics/131.2.479 PMC1205020

[46] Peakall R, Smouse PE (2007) GenAlEx V6.1, *Genetic Analysis inExcel* *Population Genetic Software for Teaching and Research*. Canberra: Australian National University Press.

[47] Hedrick PW (2005) A standardized genetic differentiation measure. Evolution 59: 1633–1638. 16329237

[48] Wright S (1943) Isolation by distance. Genetics 28: 114–138. 17247074 10.1093/genetics/28.2.114 PMC1209196

[49] Rousset F (1997) Genetic differentiation and estimation of geneflow from *F*-statisticsunder isolation by distance. Genetics 145: 1219–1228. 9093870 10.1093/genetics/145.4.1219 PMC1207888

[50] Nei M, Tajima F, Tateno Y (1983) Accuracy of estimated phylogenetic trees from molecular data. II. Gene frequency data. J Mol Evol 19: 153–170. 6571220 10.1007/BF02300753

[51] Langella O (2007) Populations 1.2.30: Population genetic software (individuals or populations distances, phylogenetic trees).France. Available: http://bioinformatics.org/~tryphon/populations/.

[52] Parks DH, Porter M, Churcher S, Wang S, Blouin C, et al (2009) GenGIS: A geospatial information system for genomic data. Genome Res 19: 1896–1904. 19635847 10.1101/gr.095612.109 PMC2765287

[53] Pritchard JK, Stephens M, Donnelly P (2000) Inference of population structure using multilocus genotype data. Genetics 155: 945–959. 10835412 10.1093/genetics/155.2.945 PMC1461096

[54] Pritchard JK, Wen XQ, Falush D (2010) Documentation for structure software: Version 2.3. Available: http://pritch.bsd.uchicago.edu/software/structure_v.2.3.1.html.

[55] Hubisz M, Falush D, Stephens M, Pritchard JK (2009) Inferring weak population structure with the assistance of sample group information. Mol Ecol 9: 1322–1332. 10.1111/j.1755-0998.2009.02591.x PMC3518025 21564903

[56] Falush D, Stephens M, Pritchard JK (2003) Inference of population structure using multilocus genotype data: Linked loci and correlated allele frequencies. Genetics 164: 1567–1587. 12930761 10.1093/genetics/164.4.1567 PMC1462648

[57] Evanno G, Regnaut S, Goudet J (2005) Detecting the number of clusters of individuals using the software STRUCTURE: a simulation study. Mole Ecol 14: 2611–2620. 10.1111/j.1365-294X.2005.02553.x 15969739

[58] Jakobsson M, Rosenberg NA (2007) CLUMPP: a cluster matching and permutation program for dealing with label switching and multimodality in analysis of population structure. Bioinformatics 23: 1801–1806. 17485429 10.1093/bioinformatics/btm233

[59] Rosenberg NA (2004) Distruct: a program for the graphical display of population structure. Mol Ecol Notes 4: 137–138.

[60] Cornuet JM, Santos F, Beaumont MA, Robert CP, Marin JM, et al (2008) Inferring population history with DIYABC: a user-friendly approach to Approximate Bayesian Computations. Bioinformatics 24: 2713–2719. 18842597 10.1093/bioinformatics/btn514 PMC2639274

[61] Cornuet JM, Ravigné V, Estoup A (2010) Inference on population history and model checking using DNA sequence and microsatellite data with the software DIYABC (v1.0). BMC Bioinformatics 11: 401. 20667077 10.1186/1471-2105-11-401 PMC2919520

[62] Estoup A, Jarne P, Cornuet JM (2002) Homoplasy and mutation model at microsatellite loci and their consequences for population genetics analysis. Mol Ecol 11: 1591–1604. 12207711 10.1046/j.1365-294x.2002.01576.x

[63] Bai WN, Liao WJ, Zhang DY (2010) Nuclear and chloroplast DNA phylogeography reveal two refuge areas with asymmetrical gene flow in a temperate walnut tree from East Asia. New Phytol 188: 892–901. 20723077 10.1111/j.1469-8137.2010.03407.x

[64] Eckert CG, Samis KE, Lougheed SC (2008) Genetic variation across species’ geographical ranges: the central–marginal hypothesis and beyond. Mol Ecol 17: 1170–1188. 18302683 10.1111/j.1365-294X.2007.03659.x

[65] Liu JF, Xiao WF, Jiang ZP, Feng X, Li XY (2005) A study on the influence of landscape fragmentation on biodiversity. Forest Research 18: 222–226 (in Chinese with English abstract)

[66] Charlesworth D (2003) Effects of inbreeding on the genetic diversity of populations. Philosophical Trans actions of the Royal Society Lond. B 358: 1051–1070. 10.1098/rstb.2003.1296 PMC1693193 12831472

[67] Petit RJ, Aguinagalde I, de Beaulieu JL, Bittkau C, Brewer S, et al (2003) Glacial refugia, Hotspots but not melting pots of genetic diversity. Science 300: 1563–1565. 12791991 10.1126/science.1083264

[68] Godbout J, Jaramillo-Correa JP, Beaulieu J, Bousquet J (2005) A mitochondrial DNA minisatellite reveals the postglacial history of jack pine *(Pinus banksiana*), a broad-range North American conifer. Mol Ecol 14: 3497–3512. 16156818 10.1111/j.1365-294X.2005.02674.x

[69] Lu ZX, Wang YH, Peng YH, Korpelainen H, Li CY (2006) Genetic diversity of *Populuscathayana*Rehd. populations in southwestern China revealed by ISSR markers. Plant Sci 170: 407–412.

[70] Tsuda Y, Sawada H, Ohsawa T, Nakao K, Nishikawa H, et al (2010) Landscape genetic structure of *Betula maximowicziana* in the Chichibu mountain range, central Japan. Tree Genet Genomes 6: 377–387.

[71] Matsui K (2010) Pollination ecology of four *Acer* species in Japan with special reference to bee pollinators. Plant Species Biol 6: 117–120.

[72] Zhang Y, Yang N, Ma Y (2003) Neotectonics in the southern part of the Taihang uplift, North China. Journal of Geomechanics 9: 313–329 (in Chinese with English abstract)

[73] Lin N, Tang J, Bian J, Yang J (1999) The Quaternary environmental evolution and the problem of desertification in Northeast Plain. Quaternary Sciences 5: 448–454 (in Chinese with English abstract)

[74] Xue X, Li H, Li Y, Liu H (2004) The new data of the uplifting of Qinling Mountains since the Middle Pleistocene. Quaternary Sci 24: 82–87 (in Chinese with English abstract)

[75] Xue X, Li W, Liu L (2002) The northward shift of Weihe River and the uplift of Qinling Mountains. Journal of Northeast University (Natural Science Edition) 32: 451–454 (in Chinese with English abstract)

[76] Buiteveld J, Vendramin GG, Leonardi S, Kamer K, Geburek T (2007) Genetic diversity and differentiation in European beech (*Fagus sylvatica* L.) stands varying in management history. For Ecol Manage 247: 98–106.

[77] Baucom RS, Estill JC, Cruzan MB (2005) The effect of deforestation on the genetic diversity and structure in *Acer saccharum* (Marsh): evidence for the loss and restructuring of genetic variation in a natural system. Conserv Genet 6: 39–50.

[78] Naydenov KD, Naydenov MK, Tremblay F, Alexandrov A, Aubin-Fournier LD (2011) Patterns of genetic diversity that result from bottlenecks in Scots Pine and the implications for local genetic conservation and management practices in Bulgaria. New For 42: 179–193.

