# Supplementary figures and images for: Genetic Structure and Hierarchical Population Divergence History of Acer mono var. mono in South and Northeast China
Source: PLoS One. 2014 Jan 31;9(1):e87187. doi: 10.1371/journal.pone.0087187 (PMC3909053; doi:10.1371/journal.pone.0087187)

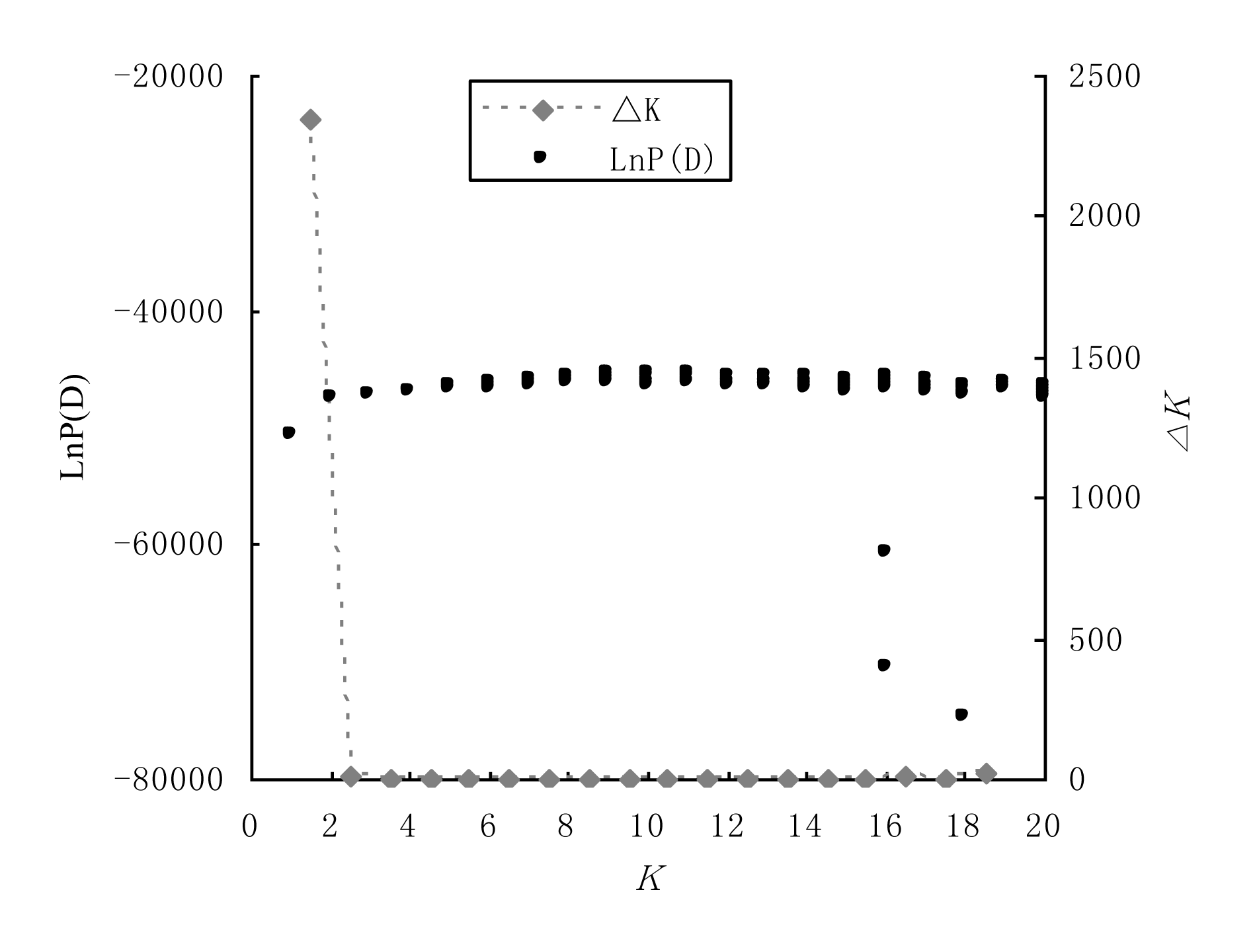

Supplement: Figure S1 — Mean values of ln P(D) and standard deviations obtained from 10 runs for each value of K = 1–20, and distributions of ΔK (Evanno et al. 2005) for K = 1–20. (TIF) [file pone.0087187.s001.tif]

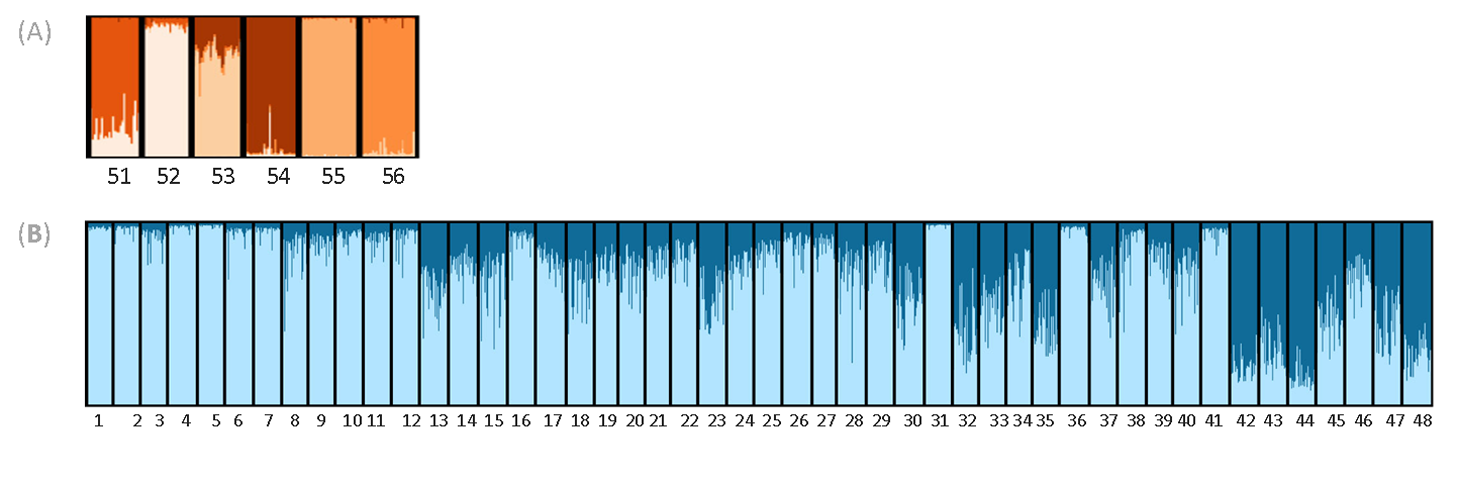

Supplement: Figure S2 — Results of additional runs of STRUCTURE analysis focused on the six SC and 48 NE populations. A, K = 6, in which the clustering corresponded to each of the six SC populations; B, K = 2 (according to the highest ΔK) for the 48 NE populations. (TIF) [file pone.0087187.s002.tif]

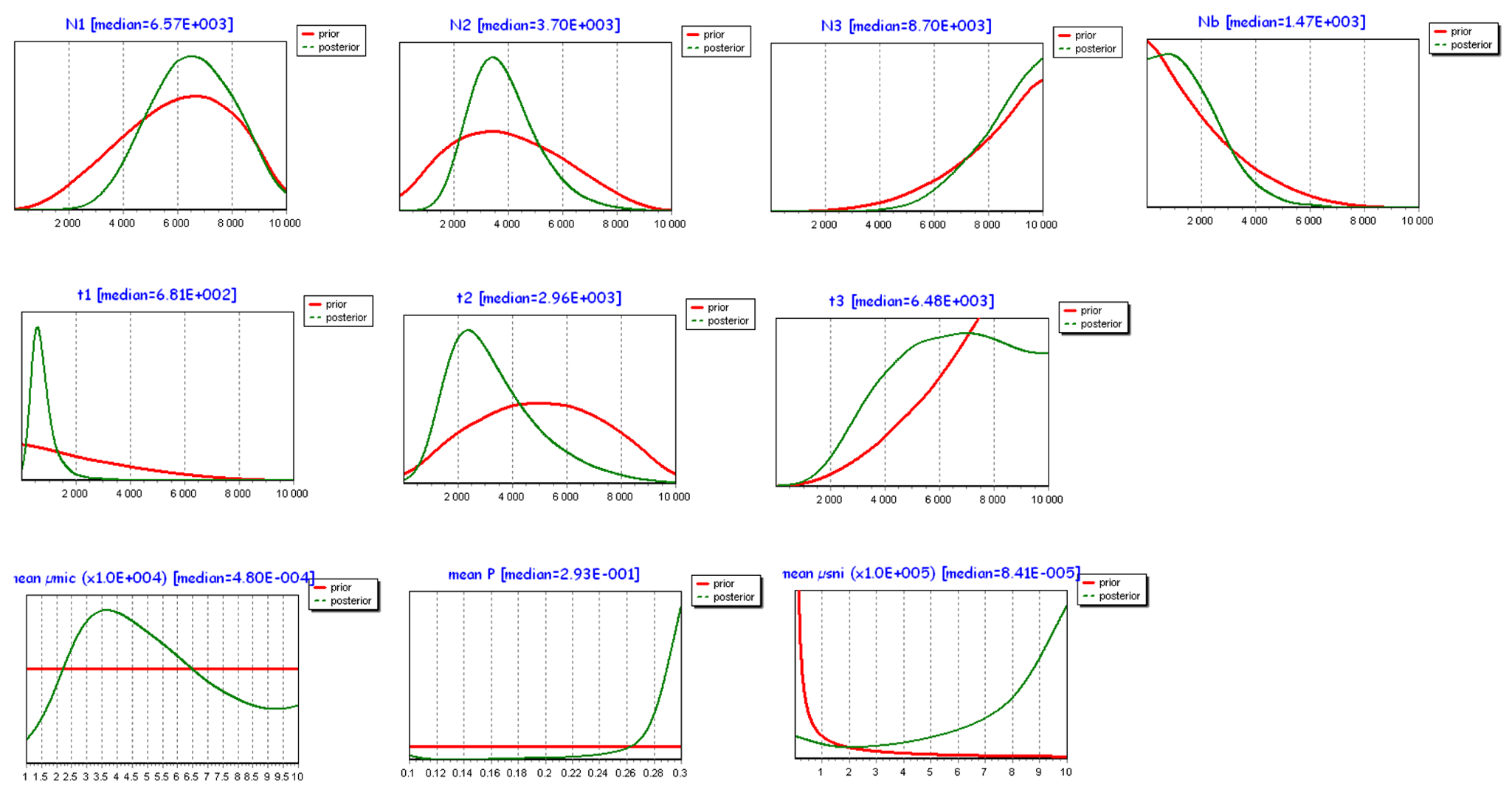

Supplement: Figure S3 — Prior and posterior distributions for each parameter obtained by DIYABC analysis of populations across the whole range. (TIF) [file pone.0087187.s003.tif]

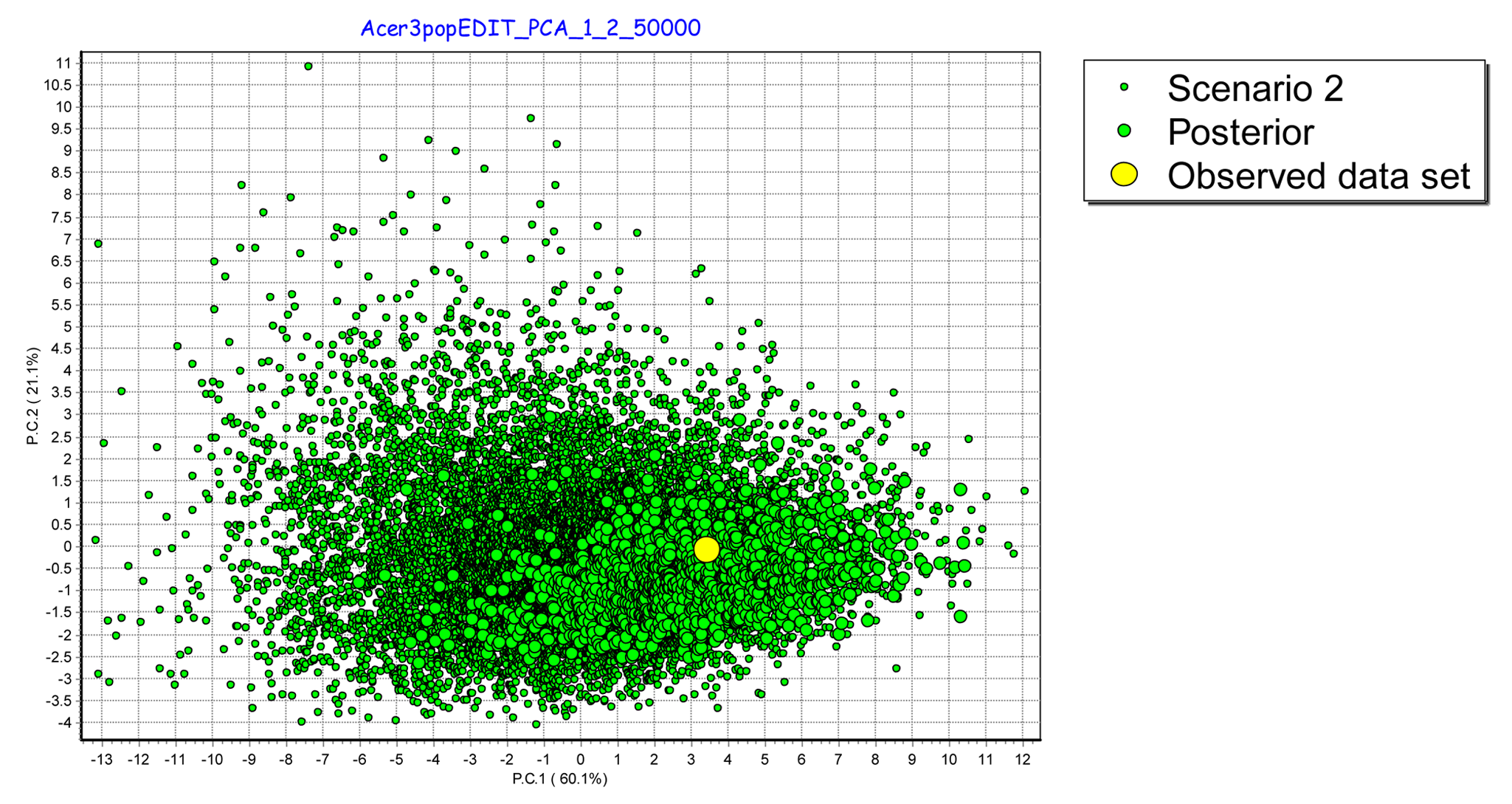

Supplement: Figure S4 — Principal Component Analysis (PCA) score plot obtained from DIYABC analysis of populations across the whole range. (TIF) [file pone.0087187.s004.tif]

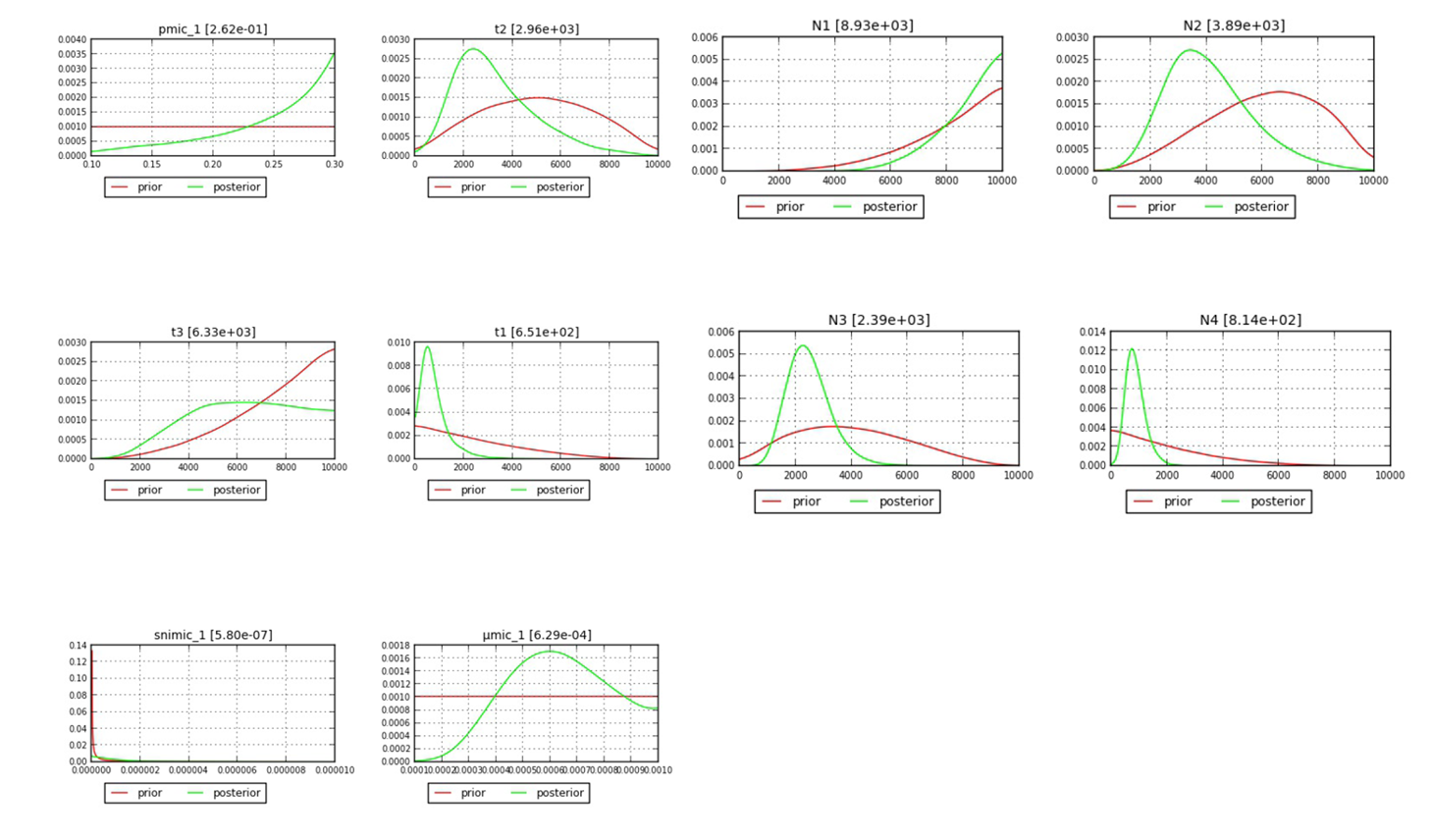

Supplement: Figure S5 — Prior and posterior distributions for each parameter obtained by DIYABC analysis of in South China populations. (TIF) [file pone.0087187.s005.tif]

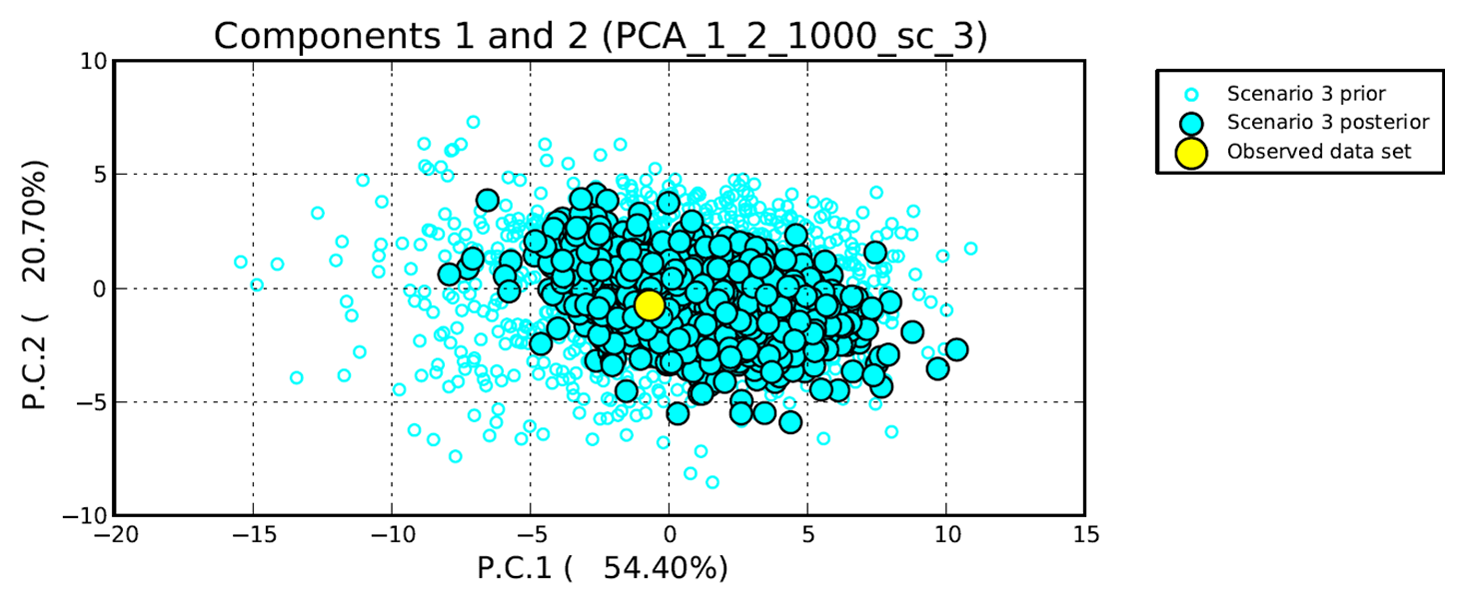

Supplement: Figure S6 — Principal component analysis (PCA) score plot obtained from DIYABC of South China populations. (TIF) [file pone.0087187.s006.tif]
