# Supplementary material for: Genetic Structure and Hierarchical Population Divergence History of Acer mono var. mono in South and Northeast China
Source: PLoS One. 2014 Jan 31;9(1):e87187. doi: 10.1371/journal.pone.0087187 (PMC3909053; doi:10.1371/journal.pone.0087187)
Supplement: Table S1 — Prior distributions of the parameters used in the whole-range DIYABC analysis. (DOC) [file pone.0087187.s007.doc]

| **Table S1** Prior distributions of the parameters used in the whole-range DIYABC analysis. | | |
| --- | --- | --- |
| Parameter | Minimum | Maximum |
| *Effective population size* |  |  |
| N1 | 10 | 10000 |
| N2 | 10 | 10000 |
| N3 | 10 | 10000 |
| Na | 10 | 10000 |
| Nb | 10 | 10000 |
| *Time scale in generations* |  |  |
| t1 | 1 | 10000 |
| t2 | 1 | 10000 |
| t3 | 1 | 10000 |
| *Admixture* |  |  |
| ra | 0.001 | 0.999 |
| *Mutation model* |  |  |
| Mean mutation rate | 1×10^-4^ | 1×10^-3^ |
| Individual locus mutation rate | 1×10^-5^ | 1×10^-2^ |
| Mean coefficient P | 1×10^-1^ | 3×10^-1^ |
| Individual locus coefficient P | 1×10^-2^ | 9×10^-1^ |
| Mean SNI mutation rate | 1×10^-8^ | 1×10^-4^ |
| Individual locus SNI rate | 1×10^-9^ | 1×10^-3^ |
