# Supplementary material for: Genetic Structure and Hierarchical Population Divergence History of Acer mono var. mono in South and Northeast China
Source: PLoS One. 2014 Jan 31;9(1):e87187. doi: 10.1371/journal.pone.0087187 (PMC3909053; doi:10.1371/journal.pone.0087187)
Supplement: Table S3 — Demographic parameters obtained from the whole-range DIYABC analysis. (DOC) [file pone.0087187.s009.doc]

| **Table S3** Demographic parameters obtained from the whole-range DIYABC analysis. | | | | | |  |  |
| --- | --- | --- | --- | --- | --- | --- | --- |
| Parameter | mean | median | mode | quantile  2.5% | quantile  5% | quantile  95% | quantile 97.5% |
| N1 | 6550 | 6570 | 6040 | 3670 | 4090 | 8930 | 9220 |
| N2 | 3860 | 3700 | 3460 | 1930 | 2140 | 6130 | 6710 |
| N3 | 8430 | 8700 | 9860 | 5530 | 6060 | 9890 | 9950 |
| t1 | 802 | 681 | 507 | 263 | 313 | 1680 | 2090 |
| t2 | 3320 | 2960 | 2150 | 1140 | 1310 | 6530 | 7340 |
| t3 | 6400 | 6480 | 7220 | 2440 | 2910 | 9630 | 9820 |
| Nb | 1670 | 1470 | 786 | 89 | 164 | 3880 | 4450 |
| Mean mutation rate_SSR | 5.15E-04 | 4.80E-04 | 3.10E-04 | 1.96E-04 | 2.20E-04 | 9.27E-04 | 9.67E-04 |
| Mean P* | 2.81E-01 | 2.93E-01 | 3.00E-01 | 1.83E-01 | 2.21E-01 | 3.00E-01 | 3.00E-01 |
| Mean mutation rate_SNI | 7.36E-05 | 8.41E-05 | 9.94E-05 | 2.30E-06 | 7.70E-06 | 9.95E-05 | 9.98E-05 |
| *The parameter of the geometric distribution used to generate multiple stepwise mutations | | | | | |  |  |
