# Supplementary material for: Genetic Structure and Hierarchical Population Divergence History of Acer mono var. mono in South and Northeast China
Source: PLoS One. 2014 Jan 31;9(1):e87187. doi: 10.1371/journal.pone.0087187 (PMC3909053; doi:10.1371/journal.pone.0087187)
Supplement: Table S4 — Comparison of summary statistics for the observed data set and posterior simulated data sets. (DOC) [file pone.0087187.s010.doc]

| **Table S4** Comparison of summary statistics for the observed data set and posterior simulated data sets. | | |
| --- | --- | --- |
| Summary statistics | observed value | P-value (simulated<observed) |
| Mean number of alleles in Pop1 | 17.4286 | 0.6930 |
| Mean number of alleles in Pop2 | 11.0000 | 0.5830 |
| Mean number of alleles in Pop3 | 20.2857 | 0.7340 |
| Mean expected heterozygosity in Pop1 | 0.7679 | 0.2665 |
| Mean expected heterozygosity in Pop2 | 0.7415 | 0.2760 |
| Mean expected heterozygosity in Pop3 | 0.8225 | 0.3285 |
| Mean number of alleles (Pop1 and Pop2) | 20.5714 | 0.7565 |
| Mean number of alleles (Pop1 and Pop3) | 26.0000 | 0.7665 |
| Mean number of alleles (Pop2 and Pop3) | 23.2857 | 0.7430 |
| Mean expected heterozygosity (Pop1 and Pop2) | 0.7789 | 0.2835 |
| Mean expected heterozygosity (Pop1 and Pop3) | 0.8453 | 0.3725 |
| Mean expected heterozygosity (Pop2 and Pop3) | 0.8368 | 0.3155 |
| F_ST_ (Pop1 and Pop2) | 0.0657 | 0.8205 |
| F_ST_ (Pop1 and Pop3) | 0.1152 | 0.8985 |
| F_ST_ (Pop2 and Pop3) | 0.1044 | 0.7415 |
| Mean index of classification (Pop1 and Pop2) | 2.3205 | 0.4770 |
| Mean index of classification (Pop1 and Pop3) | 3.2582 | 0.7370 |
| Mean index of classification (Pop2 and Pop1) | 2.2660 | 0.5955 |
| Mean index of classification (Pop2 and Pop3) | 2.9218 | 0.5310 |
| Mean index of classification (Pop3 and Pop1) | 3.6460 | 0.7325 |
| Mean index of classification (Pop3 and Pop2) | 3.3413 | 0.5185 |
