# Supplementary material for: Genetic Structure and Hierarchical Population Divergence History of Acer mono var. mono in South and Northeast China
Source: PLoS One. 2014 Jan 31;9(1):e87187. doi: 10.1371/journal.pone.0087187 (PMC3909053; doi:10.1371/journal.pone.0087187)
Supplement: Table S5 — Demographic parameters obtained from DIYABC analysis of South China populations. (DOC) [file pone.0087187.s011.doc]

| **Table S5** Demographic parameters obtained from DIYABC analysis of South China populations. | | | | | | |  |
| --- | --- | --- | --- | --- | --- | --- | --- |
| Parameter | mean | median | mode | quantile  2.5% | quantile  5% | quantile  95% | quantile  97.5% |
| N1 | 8660 | 8930 | 9900 | 5980 | 6460 | 9910 | 9960 |
| N2 | 4100 | 3890 | 3300 | 1730 | 2000 | 6970 | 7690 |
| N3 | 2480 | 2390 | 2160 | 1240 | 1380 | 3860 | 4250 |
| N4 | 866 | 814 | 790 | 331 | 393 | 1510 | 1690 |
| t1 | 828 | 651 | 461 | 177 | 222 | 2040 | 2560 |
| t2 | 3320 | 2960 | 2170 | 1080 | 1260 | 6550 | 7410 |
| t3 | 6280 | 6330 | 6370 | 2280 | 2710 | 9590 | 9800 |
| Mean mutation rate_SSR | 6.35E-04 | 6.29E-04 | 5.78E-04 | 3.02E-04 | 3.42E-04 | 9.41E-04 | 9.78E-04 |
| Mean P* | 2.47E-01 | 2.62E-01 | 3.00E-01 | 1.28E-01 | 1.42E-01 | 3.00E-01 | 3.00E-01 |
| Mean mutation rate_SNI | 1.79E-06 | 5.80E-07 | 1.05E-08 | 1.34E-08 | 1.72E-08 | 7.49E-06 | 8.73E-06 |
| *The parameter of the geometric distribution used to generate multiple stepwise mutations | | | | | |  |  |
