# Supplementary material for: Genetic Structure and Hierarchical Population Divergence History of Acer mono var. mono in South and Northeast China
Source: PLoS One. 2014 Jan 31;9(1):e87187. doi: 10.1371/journal.pone.0087187 (PMC3909053; doi:10.1371/journal.pone.0087187)
Supplement: Table S6 — Comparison of summary statistics for the observed data set and simulated posterior data sets. (DOC) [file pone.0087187.s012.doc]

| **Table S6** Comparison of summary statistics for the observed data set and simulated posterior data sets. | | |
| --- | --- | --- |
| Summary statistics | Observed value | P-value (simulated<observed) |
| Mean number of alleles in SC1 | 15.286 | 0.717 |
| Mean number of alleles in SC2 | 10.429 | 0.4595 |
| Mean number of alleles in SC3 | 7.857 | 0.6365 |
| Mean number of alleles in SC4 | 5.571 | 0.515 |
| Mean expected heterozygosity in SC1 | 0.808 | 0.1725 |
| Mean expected heterozygosity in SC2 | 0.720 | 0.1275 |
| Mean expected heterozygosity in SC3 | 0.549 | 0.0320* |
| Mean expected heterozygosity in SC4 | 0.564 | 0.172 |
| Mean number of alleles (SC1 and SC2) | 18.000 | 0.637 |
| Mean number of alleles (SC1 and SC3) | 17.857 | 0.7065 |
| Mean number of alleles (SC1 and SC4) | 16.857 | 0.7105 |
| Mean number of alleles (SC2 and SC3) | 14.143 | 0.5545 |
| Mean number of alleles (SC2 and SC4) | 11.429 | 0.4645 |
| Mean number of alleles (SC3 and SC4) | 10.571 | 0.5 |
| Mean expected heterozygosity (SC1 and SC2) | 0.802 | 0.0855 |
| Mean expected heterozygosity (SC1 and SC3) | 0.813 | 0.1145 |
| Mean expected heterozygosity (SC1 and SC4) | 0.808 | 0.1555 |
| Mean expected heterozygosity (SC2 and SC3) | 0.784 | 0.1205 |
| Mean expected heterozygosity (SC2 and SC4) | 0.733 | 0.137 |
| Mean expected heterozygosity (SC3 and SC4) | 0.730 | 0.08 |
| F_ST_ (SC1 and SC2) | 0.091 | 0.5605 |
| F_ST_ (SC1 and SC3) | 0.231 | 0.9560* |
| F_ST_ (SC1 and SC4) | 0.203 | 0.8075 |
| F_ST_ (SC2 and SC3) | 0.291 | 0.9620* |
| F_ST_ (SC2 and SC4) | 0.178 | 0.786 |
| F_ST_ (SC3 and SC4) | 0.383 | 0.9485 |
| Mean index of classification (SC1 and SC2) | 2.976 | 0.2895 |
| Mean index of classification (SC1 and SC3) | 3.743 | 0.3335 |
| Mean index of classification (SC1 and SC4) | 3.572 | 0.253 |
| Mean index of classification (SC2 and SC1) | 2.717 | 0.4175 |
| Mean index of classification (SC2 and SC3) | 3.786 | 0.3865 |
| Mean index of classification (SC2 and SC4) | 2.663 | 0.2385 |
| Mean index of classification (SC3 and SC1) | 2.956 | 0.339 |
| Mean index of classification (SC3 and SC2) | 3.218 | 0.293 |
| Mean index of classification (SC3 and SC4) | 3.574 | 0.185 |
| Mean index of classification (SC4 and SC1) | 2.701 | 0.409 |
| Mean index of classification (SC4 and SC2) | 2.069 | 0.384 |
| Mean index of classification (SC4 and SC3) | 3.587 | 0.316 |

* Significantly different from the observed data
